# Multiple factors contribute to flight behaviors during fear conditioning

**DOI:** 10.1038/s41598-023-37612-0

**Published:** 2023-06-27

**Authors:** Takafumi Furuyama, Ayana Imayoshi, Toyo Iyobe, Munenori Ono, Tatsuya Ishikawa, Noriyuki Ozaki, Nobuo Kato, Ryo Yamamoto

**Affiliations:** 1 Department of Physiology, Kanazawa Medical University, Uchinada, Ishikawa Japan; 2 Department of Functional Anatomy, Graduate School of Medical Science, Kanazawa University, Kanazawa, Ishikawa Japan

**Keywords:** Emotion, Learning and memory

## Abstract

Shifting defensive mode from one to another by the imminence of threat is crucial for survival. The transition of defensive mode from freezing to flight is observed during the modified fear conditioning, however, the flight during fear conditioning is not well characterized. To characterize the flight behaviors during the fear conditioning, we conducted experiments in male mice focusing on the influence of the context, the intensity of the unconditioned stimulus and conditioned stimulus (CS), the schedule of conditioning, and the state of the subject. Flight behaviors triggered by salient CS showed characteristics of fear-potentiated defensive behaviors depending on the conditioned context, while repetitive conditioning enhanced the expression of the flight and developed an association between the CS and the flight. The salient auditory stimulus was the primary factor to trigger flight behaviors. Also, the spaced conditioning increased the expression of flight behaviors. Taken together, the flight behavior during fear conditioning is not a simple conditioned response nor simple fear-potentiated behavior, but a complicated mixture of multiple components of defensive behaviors. The transition of defensive mode could be induced by the integration of multiple innate and learned components of fear or anxiety.

## Introduction

Appropriate expression of specific defensive behaviors under a threatening situation is essential and critical for survival. Defensive behaviors are regarded to gradually shift one from another by the imminence of threat^1–3^. These shifts of defensive behaviors are often dysregulated in anxiety disorders and post-traumatic stress disorder (PTSD) in humans^4^, and the study of animal models of defensive mode switching is essential to find new treatments for this deficit. In many cases of rodents, a typical defensive behavior switching is observed between freezing and flight behavior (i.e., jump or short darting) depending on the threat-imminence^2,3^. In the prevailing theory, freezing reflects the post-encounter threat mode, whereas flight behaviors reflect the circa-strike threat mode.

Classical fear conditioning is a widely used method to investigate mechanisms for associative fear memory acquisition^5^ and is a model of PTSD^6–8^. The extent of freezing, which is considered to be the conditioned response (CR) during classical fear conditioning, is used as the primary index to evaluate the extent of associative memory formation^5,9,10^. For a long time, freezing has been thought to be the CR during fear conditioning and it is regarded as the manifestation of the post-encounter mode^2,10^. Recently, however, flight behaviors have also been reported as CR in mice and rats during modified fear conditioning^11–13^. In these modified fear conditioning procedures, the conditioned stimulus (CS) was a serial-compound stimulus (SCS), which consist of tone and noise, and the subject exhibited the conditioned flight during the noise presentation part of SCS, irrespective of the noise-tone order. Also, rats exhibited conditioned darting, an active defensive behavior, after repetitive fear conditioning^14–16^. These reports raised the possibility that the CR could be composed of multiple behaviors and the emotional state of the subject during CS presentation is not restricted to the post-encounter mode. By contrast, another new report argued that these flight behaviors described in the reports listed above are not the CR but the fear-potentiated defensive responses^17^. These accumulating reports derived open questions, such as: are the flight behaviors during the fear conditioning a purely CR or a mixture of multiple defensive components?; what is the actual primary factor for the expression of conditioned flight?

The fine characterization of this defensive mode switching between freezing and flight behavior would contribute to developing a new clinical approach to defensive reaction disorders such as PTSD. Therefore, the present study aimed to clarify whether the fight behaviors during fear conditioning are a pure CR or a mixture of multiple components, and how multiple factors during fear conditioning affect the expression of flight behaviors. We applied a new modified fear conditioning procedure, which triggers flight behaviors in mice, not using the SCS. As described previously, CS intensity, unconditioned stimulus (US) intensity, context, training schedule, and emotional states are the factors that influence the expression of defensive behaviors in fear conditioning^18–23^. Thus, we tested various combinations of those factors during our modified fear conditionings in male mice. Some of those factors, especially the CS (tone) intensity, contributed to the expression of flight behaviors. Overall, we confirmed that the flight behaviors during fear conditioning are a mixture of fear-potentiated innate defensive behaviors and a component of CR developed with repetitive conditioning.

## Results

**Figure 1:**
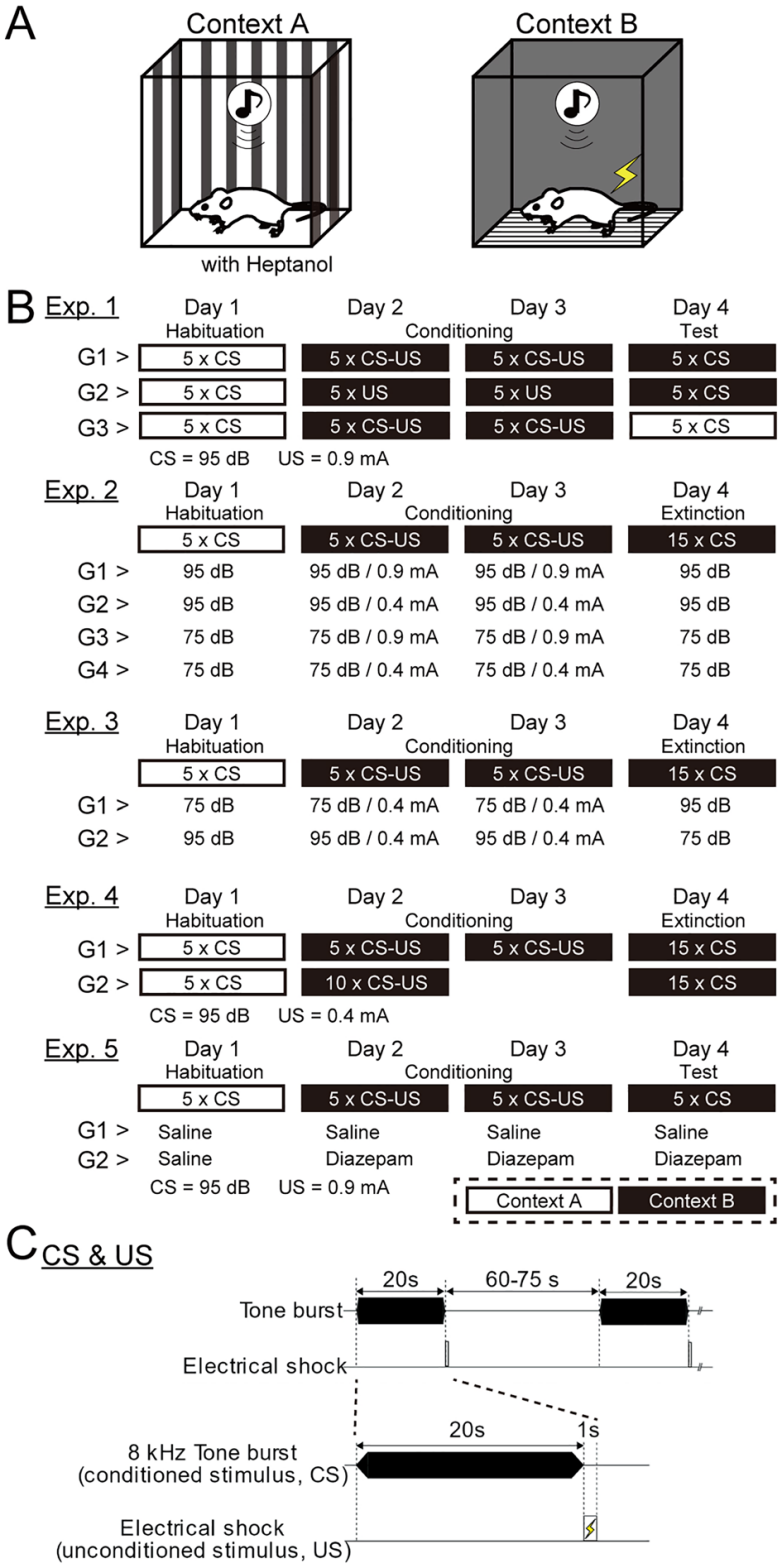
The designs of fear conditioning experiments. (**A**) Schematic drawings of experimental contexts A and B are shown. (**B**) The schedules of five experiments are listed. White and black rectangles represent the context A and B, respectively. (**C**) The composition of CS and US presentations is shown. The CS was an 8 kHz continuous tone burst (20 s) and the US (foot shock, 1 s) was delivered immediately after the CS termination. Inter-trial intervals were 60–75 s.

We conducted five fear conditioning experiments on male mice. The designs of experiments are briefly summarized in Fig. 1. Details are described in the material and methods section. All results of the statistical analysis are summarized in the supplementary information. The ‘flight behavior’ analyzed in this study is the increase of movement and jumps during CS presentations. To quantify the extent of flight behaviors, we used the total motions and the number of jumps of subjects. The ‘motion’ in this study involves all types of movement, however, the changes of ‘motion’ were almost the same as changes in the number of jumps during the CS presentations as shown in Fig. S1–3. The increase in motion was observed upon the CS presentation. Thus, the ‘motion’ during CS is used as a continuous index to evaluate the extent of flight-like movement including jumps and darting.

### Experiment 1

#### Influence of fear conditioning and conditioned context on the expression of flight behaviors

**Figure 2:**
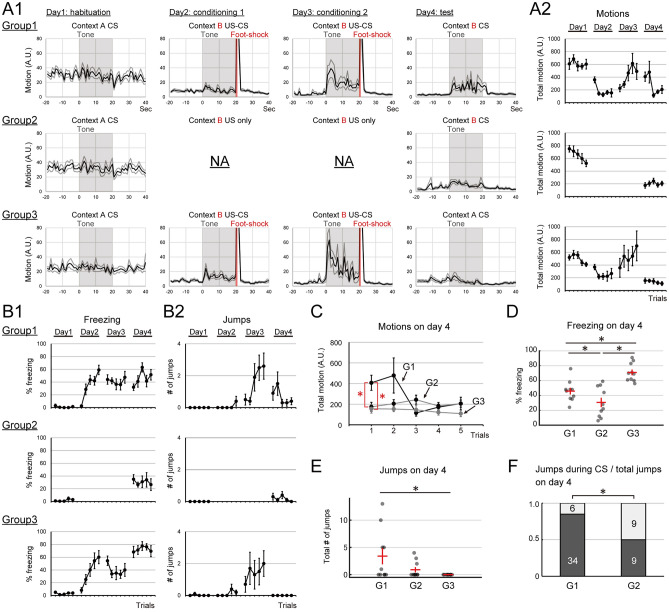
The flight behaviors during fear conditioning were fear-potentiated defensive behavior depending on the context, while the expression of the flight was enhanced with conditionings. (**A1**) Averaged motions of each condition around the CS presentation by day are shown. The gray-shaded periods represent CS presentations, and the red lines indicate US presentations. Gray lines indicate the S.E.M. of each trace. These are kept the same in all figures. On day 3, motions increased during CS presentations in G1 and G3. On day 4, the motion was increased during CS presentations in G1. (**A2**) Averaged total motions during the CS presentation of each trial are plotted. (**B1**) Averaged percentages of freezing during the CS presentation of each trial are plotted. (**B2**) Averaged jumps during the CS presentation of each trial are plotted. G1 and G2 jumped during CS presentations on day 4. (**C**) Comparison of motions on day 4. G1 moved most on the first trial than the other two groups. (**D**) Comparison of percentage freezing on day 4. There were statistically significant differences between all combinations of the three groups. The horizontal red bars indicate the averages, and the vertical red bars indicate the S.E.M. of each group. (**E**) Comparison of the total number of jumps on day 4. There were statistically significant differences between G1 and G3. The horizontal red bars indicate the averages, and the vertical red bars indicate the S.E.M. of each group. (**F**) The ratios of jumps during CS among the total jumps on day 4 are compared between G1 and G2. G1, the group conditioned with CS, jumped more during CS presentations than G2. *Indicates *p* < 0.05.

We examined the impact of fear conditioning and the conditioned context on flight behaviors. For this purpose, the expression of flight behavior was compared under three different conditions; for group 1, the contexts for conditioning (US-CS pairings) and test (CS) were the same; for group 2, the contexts for conditioning (US only, no CS) and test (CS) were the same; for group 3, the contexts for conditioning (US-CS pairings) and test were different (Fig. 1B, Experiment1). Group 1 subjects exhibited robust flight behaviors such as jump or short darting during CS presentations, especially on days 3 and 4 (Fig. 2A). Typical jumps are shown in a supplementary movie (M. 1), and a typical darting is shown in a supplementary movie (M. 2). The total motions and the number of jumps during CS presentations increased as conditioning progressed (Fig. 2A2 and B2). The extent of freezing during CS presentations was increased during day 2 and remained almost the same in the later trials (Fig. 2B1). Subjects increased movements at the onset of the CS presentation (Fig. 2A1; The 30 A.U. of ‘motion’ (not ‘total motion’) approximately corresponds to a speed of 25 cm/s) and kept showing flight behaviors during the CS presentation (Fig. 2A). These results were consistent with the characteristics of the flight behaviors of mice described in previous reports^11–13^. Subjects of Group 2 also exhibited modest flight behaviors during tone presentations on day 4 (Fig. 2B2), even though the tone was not associated with US presentations on days 2 and 3. Subjects of Group 3 exhibited robust flight behaviors similar to Group 1 on days 2 and 3 (Fig. 2A and B2), however, on day 4 in context B which was not the conditioned context, subjects did not show any flight behaviors during CS presentations (Fig. 2A and B2). Comparisons of motions during CS on day 4 revealed that Group 1 exhibited a significantly greater amount of motion than the other 2 groups in the first trial, while this behavior declined immediately as trials progressed (Fig. 2C; 2-way repeated ANOVA (factors; group and trials); interaction group * trials, *F*(8,108) = 3.34, *p* = 0.0015; group * (trial 1), *F*(2, 27) = 6.61, *p* = 0.0017; post-hoc t-test with Holm’s correction, G1 vs G2, *p* = 0.0056, G1 vs G3, *p* = 0.0039, G2 vs G3, *p* = 0.8020). Comparisons of freezing during CS on day 4 revealed that there were statistically significant differences between all three groups. Group 3 was the group that exhibited most extensive freezing (Fig. 2D; One-way ANOVA, post-hoc t-test with Holm’s correction; G1 vs G2, *p* = 0.0425; G1 vs G3, *p* = 0.014; G2 vs G3, *p* = 0.000). For the jumps on day 4, Group 1 jumped more than Group 3 (Fig. 2E; One-way ANOVA, post-hoc t-test with Holm’s correction; G1 vs G2, *p* = 0.0635; G1 vs G3, *p* = 0.0139; G2 vs G3, *p* = 0.4920). We also compared the timing of jumps between Groups 1 and 2, and found that Group 1, the group conditioned with CS (tone), jumped during tone presentations more than Group 2 which was not conditioned with tone (Fig. 2F; Fisher's exact test, G1 6/40, G2 9/18, *p* = 0.0087). All plots of each subject are shown in Fig. S1.

These results suggest that flight behaviors triggered by tone during fear conditioning are fear-potentiated defensive behaviors depending on the context and are distinct from pure CR. However, since flight behaviors are developed with repetitive contextual fear conditioning or repetitive CS-US associations, and are more associated with CS after conditioning, some aspects of flight behaviors could be regarded as CR.

### Experiment 2

#### Influence of CS intensity and US intensity on flight behaviors during fear conditioning

**Figure 3:**
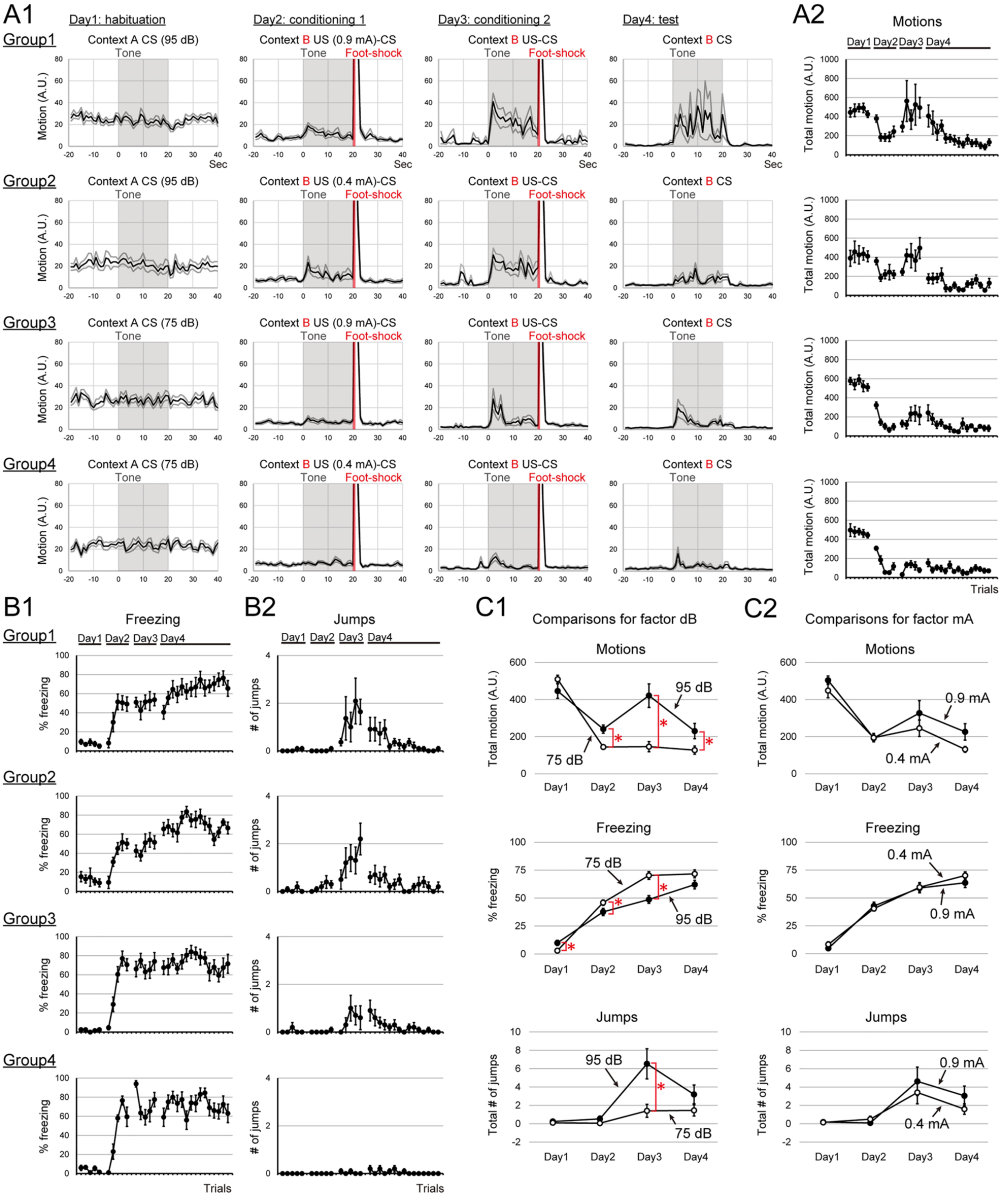
Tone intensity was the primary factor to trigger flight behaviors in the fearful context. (**A1**) Averaged motions of each condition around the CS presentation by day are shown. In general, the groups conditioned with louder CSs exhibited more movement than those conditioned with softer CSs. (**A2**) Averaged total motions during the CS presentation of each trial are plotted. (**B1**) Averaged percentages of freezing during the CS presentation of each trial are plotted. (**B2**) Averaged jumps during the CS presentation of each trial are plotted. Subjects jumped more during CS presentations on days 3 and 4 in order from G1 to G4. (**C1**) Comparisons of motions, freezing, and jumps for factor ‘dB’. Averaged total motions, averaged percentages of freezing, and averaged total numbers of jumps by days are plotted. With louder CS, subjects exhibited more flight behavior. (**C2**) Comparisons of motions, freezing, and jumps for factor ‘mA’. Averaged total motions, averaged percentages of freezing, and averaged total numbers of jumps by days are plotted. The US intensity used for the conditioning did not have a strong effect on the expression of flight behaviors. *Indicates *p* < 0.05.

We examined the impact of CS intensity and US intensity on flight behaviors. For this purpose, we compared the expression of flight behavior during fear conditioning under four different (2 CSs × 2 USs) conditions; Group1, CS = 95 dB tone, US = 0.9 mA foot shock; Group2, CS = 95 dB tone, US = 0.4 mA foot shock; Group 3, CS = 75 dB tone, US = 0.9 mA foot shock; Group 4, CS = 75 dB tone, US = 0.9 mA foot shock (Fig. 1B, Experiment 2). Group 1 subjects, conditioned with the louder CS and the stronger US (95 dB SPL tone, 0.9 mA foot shock; same parameters as Group 1 of Experiment 1), exhibited flight behaviors during CS presentations on days 3 and 4 (Fig. 3A,B), similar to Group 1 of Experiment 1. The subjects also increased movement at the onset of louder CS presentations and were fleeing during each CS presentation (Fig. 3A1). Total motions and numbers of jumps during CS presentations were highest on day 3 and kept relatively higher on day 4 (Fig. 3A,B). In contrast, the Group 4 subjects conditioned with the softer CS and the weaker US (75 dB SPL tone, 0.4 mA foot shocks) rarely exhibited flight behaviors and exhibited the freezing response (M. 3) reported in the classical fear conditioning during CS presentations (Fig. 3A,B).

To examine the effect of different US and CS intensities on the CRs, 3-way repeated ANOVA (factor; CS, US, and days) was applied to test each effect on total motions, percentages of freezing, and numbers of jumps during CS presentations averaged by days. For total motions during CS presentations, the main effect of factor ‘CS-intensity’ was statistically significant (Fig. 3C1, Motions; *F*(1, 37) = 9.10, *p* = 0.0046, (G1, G2) vs (G3, G4)), while the main effect of factor ‘US-intensity’ was not (Fig. 3C2, Motions; *F*(1, 37) = 2.68, *p* = 0.1101, (G1, G3) vs (G2, G4)). Also, the interaction between the factor ‘CS-intensity’ and factor ‘days’ was statistically significant (Fig. 3C1, Motions; *F*(3, 111) = 10.56, *p* = 0.0000). Analysis of ‘CS-intensity × days interaction’ for factor ‘CS-intensity’ revealed that the interactions were statistically significant on day 2, day 3, and day 4 (Fig. 3C1, Motions; day 2, *F*(1, 37) = 14.30, *p* = 0.0006; day 3,* F*(1, 37) = 14.64, *p* = 0.0005; day 4, *F*(1, 37) = 4.69, *p* = 0.0369). For percentages of freezing during CS presentations, the main effect of factor ‘CS-intensity’ was statistically significant (Fig. 3C1, Freezing; *F*(1, 37) = 7.49, *p* = 0.0095, (G1, G2) vs (G3, G4)), while the main effect of factor ‘US-intensity’ was not (Fig. 3C2, Freezing; *F*(1, 37) = 0.42, *p* = 0.5209, (G1, G3) vs (G2, G4)). Also, the interaction between the factor ‘CS-intensity’ and ‘days’ was statistically significant (Fig. 3C1, Freezing; *F*(3, 111) = 10.13, *p* = 0.0000). Analysis of ‘CS-intensity × days interaction’ for factor ‘CS-intensity’ revealed that the interactions were statistically significant from day 1 to day 3 (Fig. 3C1, Freezing; day 1, *F*(1, 37) = 8.87, *p* = 0.0051; day 2, F(1,37) = 4.17, *p* = 0.0483; day 3,* F*(1, 37) = 18.25, *p* = 0.0001). For number of jumps during CS presentations, the main effect of factor ‘CS-intensity’ was statistically significant (Fig. 3C1, Jumps; *F*(1, 37) = 7.51, *p* = 0.0094, (G1, G2) vs (G3, G4)), while the main effect of factor ‘US-intensity’ was not (Fig. 3C2, Jumps; *F*(1, 37) = 0.64, *p* = 0.4288, (G1, G3) vs (G2, G4)). Also, the interaction between the factor ‘CS-intensity’ and ‘days’ was statistically significant (Fig. 3C1, Jumps; *F*(3, 111) = 4.92, *p* = 0.0030). Analysis of ‘CS-intensity × days interaction’ revealed that the interactions were statistically significant on day 3 (Fig. 3C1, Jumps; day 3, *F*(1, 37) = 7.67, *p* = 0.0087). All plots of each subject are shown in Fig. S2.

These results suggested that the CS intensity (tone loudness) was the primary factor to trigger flight behaviors in the fearful context.

### Experiment 3

#### Influence of tone intensity during conditioning and test session on the expression of flight behaviors

**Figure 4:**
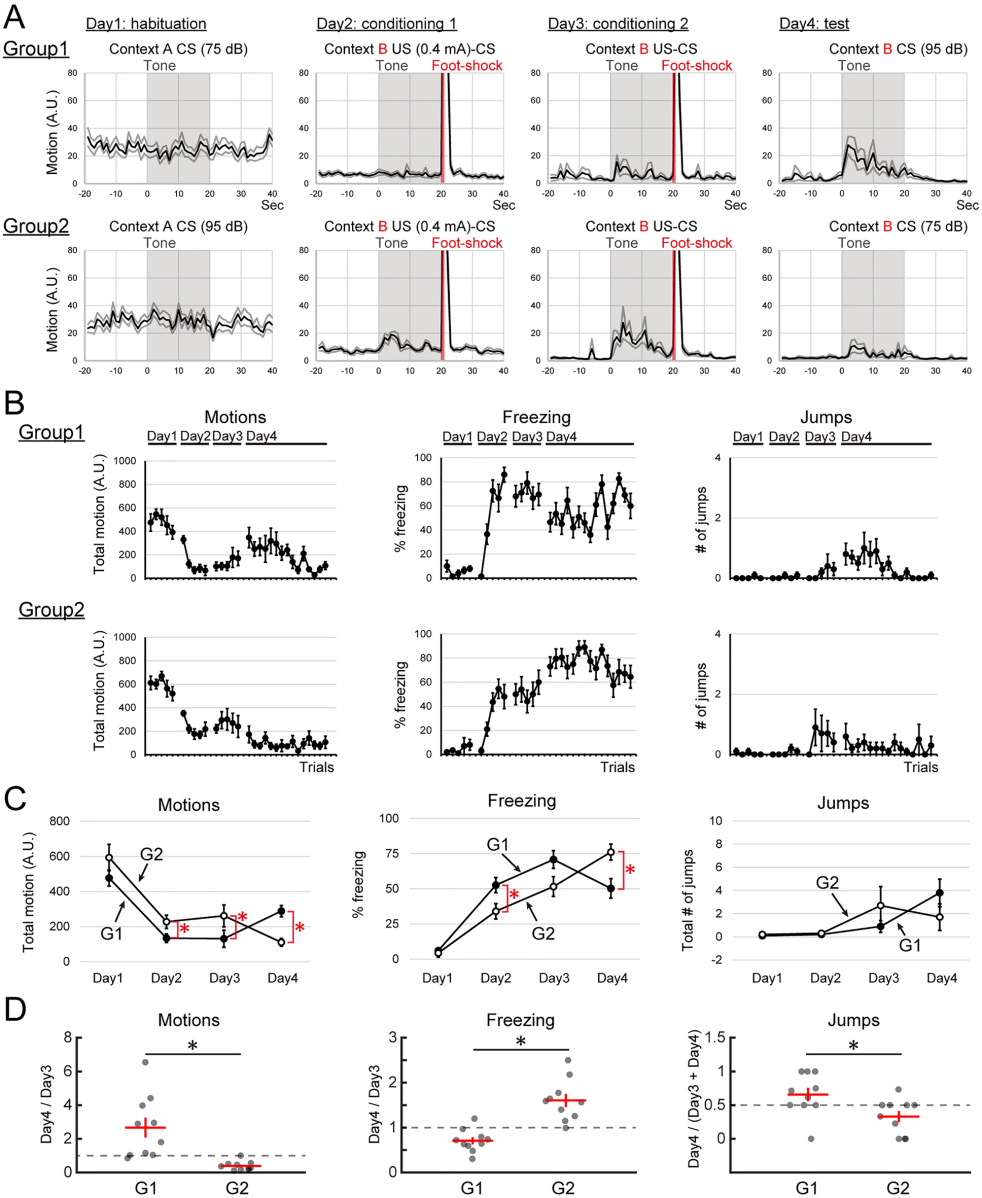
Tone intensity was the primary factor to trigger flight behaviors independent from the tone intensity used for the conditioning. (**A**) Averaged motions of each condition around the CS presentation by day are shown. G1 (upper row), G2 (lower row). (**B**) Averaged total motions, averaged percentages of freezing, and averaged jumps during the CS presentation of each trial are plotted. G1 (upper row), G2 (lower row). (**C**) Comparisons of motions, freezing, and jumps between G1 and G2. Averaged total motions, averaged percentages of freezing, and averaged total numbers of jumps by days are plotted. Each group exhibited more flight behavior on the day when the group was exposed to a louder tone. (**D**) Comparisons of ‘across-day ratio’ for motions, freezing, and jumps between G1 and G2. Each value is plotted as a gray circle. The horizontal red bars indicate the averages, and the vertical red bars indicate the S.E.M. of each group. G1 exhibited more flight behavior on day 3 than on day 4, while G2 showed the opposite. *Indicates *p* < 0.05.

We examined which CS intensity during conditioning or test sessions affected the expression of flight behaviors. We compared the expression of flight behavior during fear conditioning under two different conditions; Group1, CSs for habituation and conditioning sessions were 75 dB tone, CS for the test session was 95 dB; Group 2, CSs for habituation and conditioning sessions were 75 dB tone, CS for the test session was 95 dB (Fig. 1B, Experiment 3). On day 3, Group 2 subjects exhibited flight behaviors during CS presentations, while Group 1 subjects did not show flight behaviors. On day 4, 95 dB CS presentation during the test session elicited flight behaviors in Group 1, even though the CS intensity during conditioning sessions was 75 dB (Fig. 4A,B). In contrast, on day 4, the 75 dB CS presentation did not trigger the flight behaviors during the test session in Group 2, even conditioned with the louder CS (Fig. 4A,B).

To examine the effect of different tone intensities on flight behaviors during fear conditioning, 2-way repeated ANOVA (factor; tone-intensity and days: G1 vs G2) was applied to test each effect on total motions, percentages of freezing, and numbers of jumps during CS presentations averaged by days. For total motions during CS presentations, the main effect of factor ‘tone-intensity’ was not statistically significant (Fig. 4C, Motions; *F*(1, 18) = 0.74, *p* = 0.4066). However, the interaction between the factors ‘tone-intensity’ and ‘days’ was statistically significant (Fig. 4C, Motions; *F*(3, 54) = 8.27, *p* = 0.000). Analysis of ‘tone-intensity × days interaction’ for factor ‘tone-intensity’ revealed that the interactions were statistically significant on day 2, day 3, and day 4 (Fig. 4C, Motions; day 2, *F*(1, 18) = 7.60, *p* = 0.0139; day 3,* F*(1, 37) = 4.93, *p* = 0.0330; day 4, *F*(1, 37) = 4.45, *p* = 0.0467). For percentages of freezing during CS presentations, the main effect of factor ‘tone-intensity’ was not statistically significant (Fig. 4C, Freezing; *F*(1, 18) = 0.31, *p* = 0.5896). The interaction between the factors ‘tone-intensity’ and ‘days’ was statistically significant (Fig. 4C, Freezing; *F*(3, 54) = 14.88, *p* = 0.0000). Analysis of ‘tone-intensity × days interaction’ for factor ‘tone-intensity’ revealed that the interactions were statistically significant on day 2 and day 4 (Fig. 4C, Freezing; day 2, *F*(1, 18) = 5.80, *p* = 0.0279; day 4,* F*(1, 37) = 8.28, *p* = 0.0111). For numbers of jumps during CS presentations, the main effect of the factor ‘tone-intensity’ was not statistically significant (Fig. 4C, Jumps; *F*(1, 18) = 0.00, *p* = 0.9942). Also, the interaction between factors ‘tone-intensity’ and ‘days’ was not statistically significant (Fig. 4C, Jumps; *F*(3, 54) = 2.17, *p* = 0.0921). Furthermore, we compared the ‘across-days ratio’ of motion, freezing, and jumps across day 3 and day 4 in each individual to examine the effect of flipping tone intensity. In all three ‘across-days ratios’, there were statistically significant differences between Groups 1 and 2 (Fig. 4D; G1 vs G2; permutation test; Motion, 2.67 ± 0.59 vs 0.39 ± 0.59, *p* < 0.0000; Freezing, 0.71 ± 0.08 vs 1.60 ± 0.15, *p* < 0.0000; Jumps, 0.66 ± 0.10 vs 0.33 ± 0.08, *p* = 0.0216). All plots of each subject are shown in Fig. S3.

These results also support the idea that CS intensity is the primary factor to trigger flight behaviors, and the louder CS presentation during the conditioning is not essential for the expression of flight behaviors.

### Experiment 4

#### Comparison between single-day conditioning and 2 days conditioning on the expression of flight behaviors

**Figure 5:**
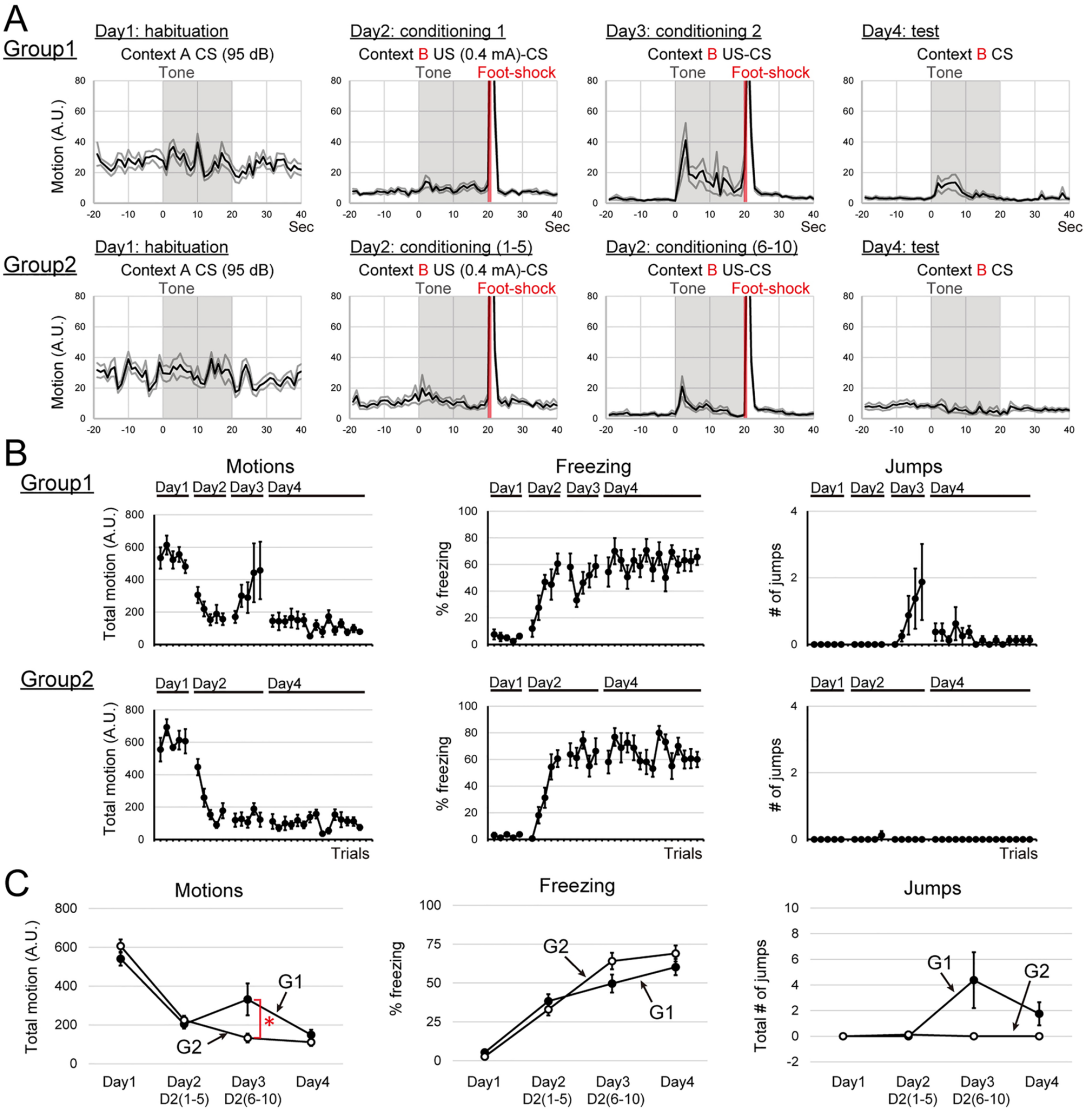
Across days conditionings enhanced the expression of flight behavior. (**A**) Averaged motions of each condition around the CS presentation by day are shown. G1 (upper row), G2 (lower row). (**B**) Averaged total motions, averaged percentages of freezing, and averaged jumps during the CS presentation of each trial are plotted. G1 (upper row), G2 (lower row). (**C**) Comparisons of motions, freezing, and jumps between G1 and G2. Averaged total motions, averaged percentages of freezing, and averaged total numbers of jumps by days are plotted. G2, the one-day conditioned group, exhibited fewer flight behaviors than G1. *Indicates *p* < 0.05.

We tested whether the number of conditioning days affects the expression of flight. We compared the expression of flight behavior during fear conditioning under two different training schedules; Group1, the conditioning sessions consisted of two days, 5 US-CS pairings each; Group 2, the conditioning session was a single day, 10 US-CS parings (Fig. 1B, Experiment 4). Group 1 subjects exhibited flight behaviors during CS presentations as trials (6th to 10th trials of the conditioning) progressed on day 3, while Group 2 subjects did not show flight behaviors during 6th to 10th trials of the conditioning, the latter half of conditioning (day 2; Fig. 5A,B). On day 4, CS presentations elicited flight behaviors in Group 1, but not in Group 2 (Fig. 5A,B).

To examine the effect of days conditioned on flight behaviors, 2-way repeated ANOVA (factor; training schedule and days: G1 vs G2) was applied to test each effect on total motions, percentages of freezing, and numbers of jumps during CS presentations averaged by days. For total motions during CS presentations, the main effect of factor ‘schedule’ was not statistically significant (Fig. 5C, Motions; *F*(1, 14) = 0.77, *p* = 0.3929). The interaction between factors ‘schedule’ and ‘days’ was statistically significant (Fig. 5C, Motions; *F*(3, 42) = 5.47, *p* = 0.0029). Analysis of ‘schedule × days interaction’ for factor ‘schedule’ revealed the interaction was statistically significant on day 3 (Fig. 5C, Motions; day 3,* F*(1, 14) = 4.35, *p* = 0.0483). The percentages of freezing during CS presentations, the main effect of factor ‘schedule’ was not statistically significant (Fig. 5C, Freezing; *F*(1, 14) = 0.81, *p* = 0.3853). The interaction between factors ‘schedule’ and ‘days’ was statistically significant (Fig. 5C, Freezing; *F*(3, 42) = 3.17, *p* = 0.0368). Analysis of ‘schedule × days interaction’ revealed that the *F* value for factor ‘schedule’ was relatively large on day 3 (Fig. 5C, Freezing; day 3,* F*(1, 14) = 3.38, *p* = 0.0917). For number of jumps during CS presentations, the main effect of factor ‘schedule’ was statistically significant (Fig. 5C, Jumps; *F*(1, 14) = 5.39, *p* = 0.0280). Analysis of ‘schedule × days interaction’ for factor ‘days’ revealed the interaction was statistically significant in Group 1, not in Group 2 (Fig. 5C, Jumps; G1, *F*(3, 42) = 6.56, *p* = 0.0267; G2, *F*(3, 42) = 0.01, *p* = 0.0927). All plots of each subject are shown in Fig. S3.

These results suggest that repetitive conditionings across days enhance the expression of flight behaviors and are consistent with the importance of the conditioned context as confirmed in Experiment 1.

### Experiment 5

#### Effect of Diazepam, an antianxiety drug, on the expression of flight behaviors.

We tested whether the fear states affect the expression of flight behaviors or not. For this purpose, we examined the effect of Diazepam, an antianxiety drug^24^, on flight behaviors. First, we confirmed the effect of Diazepam (1.2 mg/kg) on locomotion and anxiety with the elevated plus maze test. With this dose of Diazepam, subjects increased the time spent in the open arm than the saline control group, while the distances traveled were not different between the two groups in the elevated plus maze test (Fig. S4; saline (n = 4) vs Diazepam (n = 4); permutation test; percent time spent in the open arm, 4.5 ± 1.1% vs 28.0 ± 7.7%, *p* = 0.0242; distance traveled, 1253 ± 88 vs 1732 ± 369, *p* = 0.3224).

**Figure 6:**
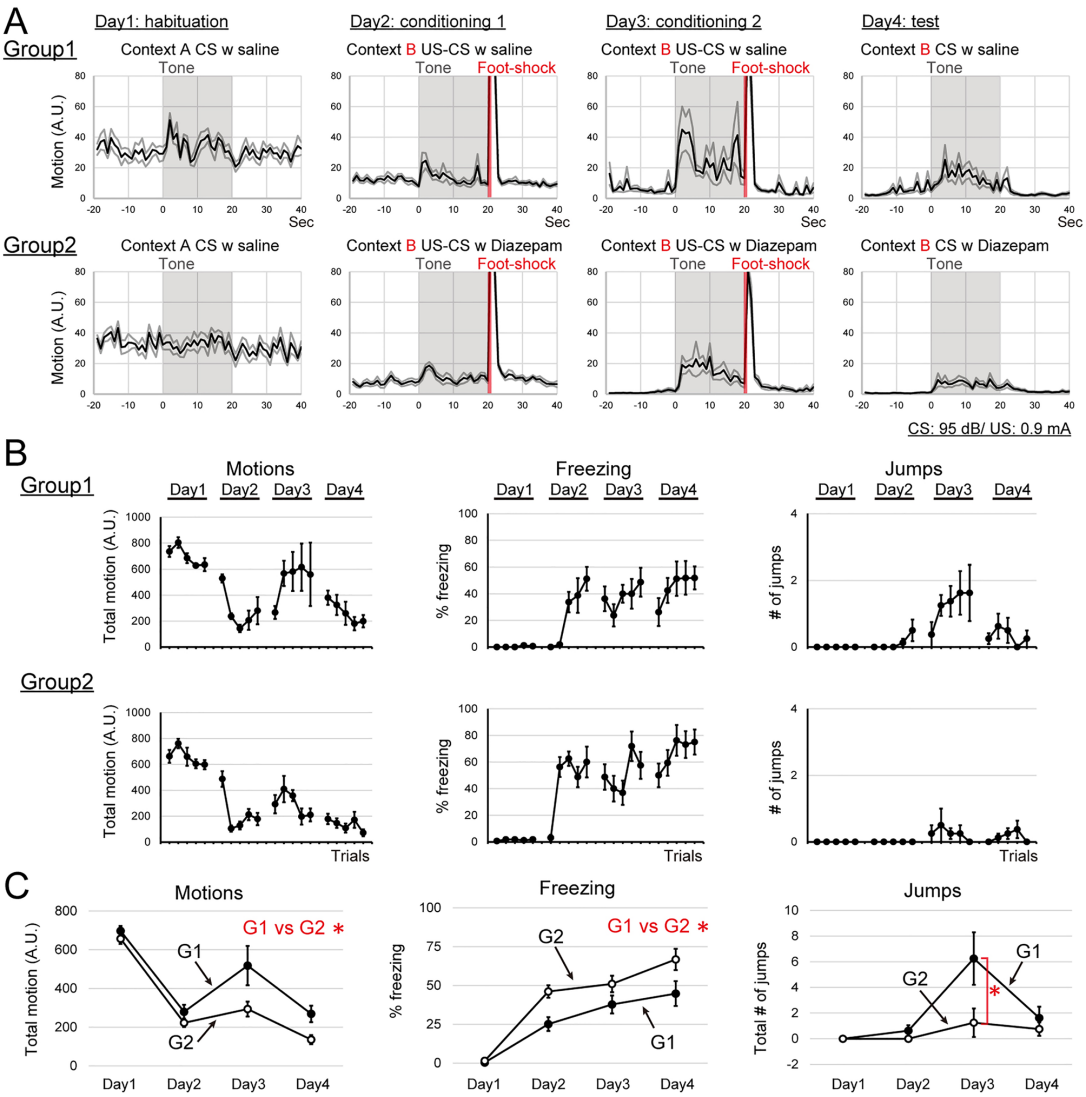
Diazepam, an anti-anxiety drug, attenuated the expression of flight behavior. (**A**) Averaged motions of each condition around the CS presentation by day are shown. G1 (upper row), G2 (lower row). (**B**) Averaged total motions, averaged percentages of freezing, and averaged jumps during the CS presentation of each trial are plotted. G1 (upper row), G2 (lower row). (**C**) Comparisons of motions, freezing, and jumps between G1 and G2. Averaged total motions, averaged percentages of freezing, and averaged total numbers of jumps by days are plotted. G2, the Diazepam-treated group, exhibited fewer flight behaviors than G1. *Indicates *p* < 0.05.

Next, we tested the effect of Diazepam (1.2 mg/kg) on the expression of flight behaviors (Fig. 1B, Experiment 5). For the first day, Groups 1 and 2 received saline injections, 30 min before the start of the session. From day 2 to day 4, Group 1 received saline, and Group 2 received Diazepam, 30 min before the start of each session. Group 1 subjects exhibited flight behaviors during CS presentations from the fourth or fifth trials on day 2 and the flight behaviors were developed as trials progressed on day 3 (Fig. 6A,B). The characteristic of flight behaviors was similar to those of Experiment 1 Group 1 and Experiment 2 Group 1 (Fig. 6A). Group 2 subjects, the Diazepam-treated group, exhibited fewer flight behaviors (Fig. 6A,B).

To examine the effect of Diazepam on flight behaviors, 2-way repeated ANOVA (factor; drug and days: G1 vs G2) was applied to test each effect on total motions, percentages of freezing, and numbers of jumps during CS presentations averaged by days. For total motions during CS presentations, the main effect of factor ‘drug’ was statistically significant (Fig. 6C, Motions; *F*(1, 14) = 9.71, *p* = 0.0073). The interaction between factors ‘drug’ and ‘days’ was not statistically significant (Fig. 6C, Motions; *F*(3, 42) = 1.77, *p* = 0.1666). The percentages of freezing during CS presentations, the main effect of factor ‘drug’ was statistically significant (Fig. 6C, Freezing; *F*(1, 14) = 14.28, *p* = 0.0025). The interaction between factors ‘drug’ and ‘days’ was not statistically significant (Fig. 6C, Freezing; *F*(3, 42) = 1.84, *p* = 0.1506). For number of jumps during CS presentations, the main effect of factor ‘drug’ was statistically significant (Fig. 6C, jumps; *F*(1, 14) = 4.59, *p* = 0.0444). The interaction between factors ‘drug’ and ‘days’ was statistically significant (Fig. 6C; *F*(3, 42) = 3.59, *p* = 0.0139). Analysis of ‘drug × days interaction’ for the factor ‘drug’ revealed that the interaction was statistically significant on day 3 (Fig. 6C, jumps; day 3,* F*(1, 14) = 4.59, *p* = 0.0338). Analysis of the interaction for the factor ‘day’ revealed that the interaction was statistically significant in Group 1, not in Group 2 (Fig. 6C, jumps; G1, *F*(3, 42) = 11.06, *p* = 0.0014; G2, *F*(3, 42) = 0.52, *p* = 0.1436). All plots of each subject are shown in Fig. S3.

These results implied that the internal states modulated with the antianxiety drug might alter the expression of flight behaviors.

## Discussion

The present study confirmed that flight behaviors (an increase of movement and jumps, in this study) during modified fear conditionings are a mixture of fear-potentiated defensive behaviors and conditioned responses developed with repetitive conditioning in male mice. For triggering the flight behaviors, a salient tone is required, while SCS or noise presentation is not necessary. Furthermore, our results raised the possibility that spaced conditioning induces metaplastic changes in the emotional state which modulate the defensive mode. The present study clarifies our understanding of the neural and behavioral mechanisms for processing threat imminence. The transition of defensive mode could be induced by the integration of multiple innate and learned components of fear or anxiety.

### The flight behaviors during fear conditioning could be regarded as fear-potentiated defensive behavior, while the expression of flight is enhanced with repetitive conditioning

We have demonstrated that a salient CS stably triggered flight behaviors (an increase of movement and jumps) in male mice during fear conditioning (Experiment 1, Group 1). To evaluate the extent of flight expression, we use the indexes ‘motion’ and ‘jump’. ‘Jump’ is a typical flight behavior, while ‘motion’ involves all types of movement, which is not necessarily flight behavior. However, we think ‘motion’ is a useful index to evaluate flight at least in the present experiment, since the changes in ‘motion’ in this study almost corresponded with the changes in the number of jumps as shown in Figs. 1, 2, 3. Characteristics of the flight behavior and the rapid changes of motion during CS presentations were consistent with previous studies that reported flight behaviors in mice^11,12,17^. We also demonstrated that a salient tone presentation, which was not associated with a foot shock, triggered flight behaviors after repetitive contextual fear conditioning (Experiment 1, Group 2), and the conditioned context was essential for the expression of flight behaviors (Experiment 1, Group 3). These results suggest that the flight behaviors triggered by CS during fear conditioning could be regarded as a fear-potentiated defensive behavior depending on the context, and consistent with the idea proposed by Trott and colleagues^17^. Moreover, we confirmed that the expression of flight behaviors is enhanced by increasing the number of contextual conditioning sessions (Experiment 1, Group 2), CS and US pairings (Experiment 1, Groups 1 and 3), and spacing of conditioning trials across days (Experiment 4). These results suggest that the repetitive conditioning develops the conditioned state which shifts the defensive mode, and the flight during fear conditioning is not a simple fear-potentiated behavior. This conditioned state, which is associated with the context or CS, could represent emotional states similar to fear or anxiety, but would not be a simple emotional state that triggers direct behaviors. Totty and colleagues reported that rats exhibited the flight during CS presentations in an unconditioned context, after three days of conditioning, which would be robust conditioning^13^. While we could not replicate their result in our similar experiment (Experiment 1, Group 3), the comparison between Groups 1 and 2 of Experiment 1 revealed that the repetitive conditionings developed an association between flight behavior and CS. We assume that if the CS-conditioned state (CS-dependent fear potentiation, in other words) is robust enough to shift the defensive mode, CS presentation without the conditioned context could increase the chance of flight behavior expression. The fast decline of fight behavior expression during test sessions (day 4 of each experiment) also supports these ideas. If the flight behavior is a simple CR similar to freezing, the response should be more stably expressed during repetitive CS presentations in the test session. Note that mice did not exhibit the enhancement of flight behavior expression after the association between CS (tone) and US in a previous report^17^. Probably the difference between the strain of mice (Trott et al. 2022. C57BL/6N; ours, C57BL/6 J) would explain this dissociation between our and their results. Several studies reported that the C57BL/6N strain is more anxious and susceptible to stress than the C57BL/6 J strain^25–28^. Thus, the fear state might be saturated during contextual fear conditioning, and there might not be room for enhancement. Taken together, the flight behaviors during fear conditioning are fear-potentiated defensive behaviors developed with repetitive fear conditioning, which are only induced after the conditioning and are associative with non-aversive stimuli. We conclude that the flight during fear conditioning is not a simple CR nor fear-potentiated behavior, but a complicated mixture of multiple components of defensive behaviors.

The results of Diazepam in Experiment 5 would support our argument that a conditioned emotional state or a conditioning-potentiated fear is involved in the expression of flight behavior. Diazepam treatment reduced the flight expression and interestingly increased the freezing response during CS presentation. The reduction of fear or anxiety would diminish shifts in the defensive mode from post-encounter to circa strike. This result might be considered inconsistent with previous reports that demonstrated that Diazepam reduced freezing^29–31^. However, given that freezing would be attenuated by flight behavior as fear increases, the reduction of fear causes the increase of freezing when the subject feels extreme fear, therefore the result of Diazepam in Experiment 5 does not contradict previous findings. Thus, the extent of freezing would not be a good index to evaluate the extent of fear or associative fear memory. We used Diazepam as an antianxiety drug and interpreted that Diazepam could alter the internal states of animals, while it is well known that Diazepam also acts as a sedative drug that reduces the locomotor activity of subjects. At least, in the elevated plus maze test, we confirmed that the dose of Diazepam in this experiment (1.2 mg/kg) did not affect the locomotion activity, while it increased the open arm exploration. Thus, the primary effect of Diazepam in our experiment would be the antianxiety effect, not the sedative effect, and the antianxiety effect would be the effect that modulated the expression of flight behaviors. Of course, the possibility that the repeated administration of Diazepam would have a robust sedative effect should still be considered. More fine experiments to investigate the involvement of internal states in the expression of flight behaviors need to be conducted in future studies.

Some reports have demonstrated the dimorph of the flight behavior in rats^14–16^ and mice^32^, while a couple of reports did not reproduce the sex difference of the flight behavior in rats^13^ and mice^17^. Thus, it remains controversial whether there is a sex difference in the flight behaviors of mice or not. In the present study, we only focused on the flight behaviors of male mice, and this is the limitation of this study for understanding more general features of the flight behavior. Further investigations must be required to elucidate the dimorphic effect of fear on active defensive behaviors.

### A salient tone triggers flight behavior in the fearful context

We demonstrated that the salient tone (95 dB tone burst) was the primary factor to trigger flight behaviors in the fear-conditioned context. The soft CS (75 dB tone) triggered fewer flight behaviors than the louder one (Experiment 2). US intensity or CS intensity during the conditioning was not the essential factor for the expression of flight behaviors (Experiments 2 and 3). This finding suggests that the salient auditory signal is the key factor to trigger flight behaviors as fear conditioning progresses. Consistent with this idea, in the previous reports, the noise part of the SCS, a more salient compound, triggered the flight behavior^11,12,17^. Several reports listed below support the idea that noise is perceived as a more salient signal than pure tone at the same level of dB SPL. The best frequency for the auditory perception of C57BL6J mice is around 12–16 kHz^33^, and broadband noise contains these frequencies. Moreover, noise presentations recruit more neural activity than the same dB tone^34^. Taken together with our results, a more salient auditory signal seems to represent a more proximal threat that facilitates the expression of circa-strike flight behaviors in a fearful context.

Meanwhile, the salient auditory signal itself can induce defensive behaviors in mice and rats. Rats avoid noise and noise presentation causes a reduction of dopamine in the nucleus accumbens^35^. Auditory looming triggers active defense behaviors in mice^36^. And as it has been well known, mice or rats show the auditory startle by salient auditory stimuli^37,38^. This startle is potentiated with fear conditioning and regarded as an alpha response of defensive behavior^38–40^. The startle shares a lot of similarities with the flight behaviors described in this study and previous reports^11,12,17^. For example, both the startle and the flight are triggered by salient auditory stimuli without any conditioning. Also, fear conditioning potentiated the amplitude of the startle and the expression of flight behaviors. And Diazepam suppresses acoustic startle^41,42^ and the expression of flight behaviors (Experiment 5). However, we confirmed that the flight behaviors were observed during the entire CS presentation period in the test session (i.e., Fig. 2A1 day 4; Fig. 3A1 day 4), and also the flight developed after contextual conditioning that lacked any presentation of salient stimuli (Experiment 1, Group 2, Fig. 2F). Moreover, the flight behaviors were not triggered even by a loud noise presentation during the habituation session (day 1 of all experiments). These findings clearly indicate that the flight behaviors during conditioning are different from a simple alpha response such as a non-associative acoustic startle. Thus, as discussed in the previous section, we propose that the flight behaviors in this experiment are a mixture of fear-potentiated behavior, similar to the alpha-response, and a component of CR expressed under a fearful state.

For a long time, freezing, a passive defensive behavior, is regarded as the defensive behavior when the threat is predictable, however, the present study and previous studies^11–17^ have shown that rodents exhibit ‘flight’, active defensive behaviors, during modified fear conditioning. Classical fear conditioning in rodents was often used as a model of PTSD^6–8^, while it does not cover panic or marked active reactions, the symptoms of PTSD^4^. The flight behaviors shown in recent studies are similar to panic or exaggerated active defensive behaviors, thus the modified fear conditionings used in recent studies would be a better test to investigate PTSD and other anxiety disorders. By using these new models, fine studies to elucidate the neural correlates for symptoms of PTSD would be conducted in the future.

## Materials and methods

Experiments were performed in accordance with the guiding principles of the Physiological Society of Japan and were approved by the Animal Care Committee of Kanazawa Medical University (2021-32). All animal experiments complied with the ARRIVE guidelines and were carried out in accordance with National Institutes of Health guide for the care and use of Laboratory animals (NIH Publications No. 8023, revised 1978).

### Subjects

Male C57BL/6 J mice (10–16 weeks old, n = 131) were used. In this study, we focused on the flight behaviors of males. Subjects were group-housed (3–4 per cage) in plexiglass cages (20 × 40 × 17 cm) under a 12 h light/dark cycle. All subjects were bred and maintained in the laboratory at 22–23 °C under a 12 h light/dark cycle. Food and water were available ad libitum.

### Apparatus

A fear conditioning chamber (25 × 25 × 25 cm, LE116, Panlab S.L.U., Barcelona, Spain) was used and enclosed in a sound-attenuating box (67 × 53 × 55 cm). A scramble shocker (LE 100-26, Panlab S.L.U., Barcelona, Spain) was connected to a grid floor composed of stainless-steel rods. The floor of the test chamber was placed on a transducer for the detection of vibration (LE 111, Panlab S.L.U., Barcelona, Spain). The signal of the transducer was carried to a sound card (UMC202, Behringer, Willich, Germany) with an 8 kHz sampling frequency for recording the vibration of behavior. A speaker (FT17H, Fostex, Tokyo, Japan) and a CMOS camera (CMS-V43BK, Sanwa Supply Inc., Okayama, Japan) were placed on the ceiling. All acoustic stimuli were amplified (TA-F500, Sony, Tokyo, Japan), and the overall amplitudes of each stimulus were digitally modified and calibrated (with a 1/4-inch microphone: type 4156N, ACO, Tokyo, Japan) to yield sound pressure levels (SPL, re: 20 μ Pa) at the 5 cm front of the speaker. The experimental box was illuminated with an overhead white light-emitting diode (240 lx). The chamber was cleaned with 70% alcohol following each trial of behavioral tests.

The elevated plus maze was composed of open arms (50 mm width, 375 mm length), closed arms (50 mm width, 375 mm length, 150 mm opaque wall), and a center area (Fig. S4A). The test was conducted in a dark circumstance (10 lx) and recorded with an infrared camera (ELP-USBFHD05MT-KL36IR-J, Ailipu Technology, Shenzhen, China).

### Modified fear conditioning procedures

Each subject was singly housed in a plexiglass cage (14 cm × 21 cm × 12 cm) for at least 3 days before undergoing the fear conditioning. On all testing days, each subject was placed in the fear conditioning chamber for 3 min before the presentation of the first CS. Two contexts (A and B) were used for the experiments. For context A, the appearance walls were black and white stripes, the floor was white smooth plastic board. The walls and floor were wiped with Heptanol (1%) odor (Fig. 1A). For context B, the appearance walls were entirely black, the floor was a grid floor, and no specific odor was presented (Fig. 1A). Fear conditioning procedures consisted of habituation (1 day, 5 trials), conditioning (2 days, 5 trials each), and test/extinction sessions (1 day, 5 or 15 trials; Fig. 1B). The intertrial intervals varied between 60 and 75 s (Fig. 1C). During the conditioning session, the US (1 s, 0.4 or 0.9 mA) was presented immediately after the termination of CS (8 kHz, 20 s, 75 or 95 dB SPL) as shown in Fig. 1C. Five CS-US pairs were delivered in a conditioning day. The subject was left in the context for 1 min after the end of the 5th foot-shock termination and then was returned to the home cage. Each subject underwent a fear conditioning procedure one time.

*Experiment 1* This experiment was conducted to examine the influence of fear conditioning and conditioned context on the expression of flight behaviors. A CS (95 dB SPL) and a US (0.9 mA) were used in this experiment. On day 1, mice were exposed to 5 CS-alone trials in context A. For Groups 1 (n = 10) and 3 (n = 10), mice were conditioned with 5 CS-US trials in context B on days 2 and 3. For Group 2 (n = 10), mice were conditioned with 5 US presentations in context B on days 2 and 3. On day 4, Groups 1 and 2 were exposed to 5 CS-alone trials for the recall session in context B. Group 3 was tested in context A.

*Experiment 2* This experiment was conducted to examine the influence of CS intensity and US intensity on the expression of flight behaviors during fear conditioning. Two different intensities for both conditioned stimulus (CS) and unconditioned stimulus (US) were used in this experiment. The CS was continuous tones (8 kHz, 20 s, 95 or 75 dB) and the US was foot-shock (1 s, 0.9 or 0.4 mA). In this experiment, four groups were used (Group 1, CS = 75 dB and US = 0.4 mA, n = 11; Group 2, CS = 75 dB and US = 0.9 mA, n = 10; Group 3, CS = 95 dB and US = 0.4 mA, n = 10; Group 4, CS = 95 dB and US = 0.9 mA, n = 10). On day 1, mice were exposed to 5 CS-alone trials in context A. On days 2 and 3, mice were conditioned with 5 CS-US trials in context B. On day 4, mice were exposed to 15 CS-alone trials for the extinction session in context B.

*Experiment 3* This experiment was conducted to determine which CS intensity during conditioning or test sessions affected the expression of flight behaviors. Two intensities (95 and 75 dB) of CS and one intensity (0.4 mA) of US were used in this experiment. Two groups were tested for fear conditioning. On day 1, mice were exposed to 5 CS-alone trials in context A. On days 2 and 3, mice were conditioned with 5 CS-US trials in context B. On day 4, mice were exposed to 15 CS-alone trials for the extinction session in context B. For Group 1 (n = 10), CS intensities from days 1–3 were 75 dB, and the intensity on day 4 was 95 dB. For Group 2 (n = 10), CS intensities from days 1–3 were 95 dB, and the intensity on day 4 was 75 dB.

*Experiment 4* This experiment was conducted to examine whether there is a difference between spaced and massed conditioning on the expression of flight behaviors. A CS (95 dB) and a US (0.4 mA) were used. In this experiment, two groups were tested for fear conditioning. On day 1, mice were exposed to 5 CS-alone trials in context A. On days 2 and 3, Group 1 (n = 8) was conditioned with 5 CS-US trials in context B. Group 2 (n = 8) was exposed to 10 CS-US trials on day 2. On day 4, both groups were exposed to 15 CS-alone trials in context B.

*Experiment 5* This experiment was conducted to examine the effect of Diazepam, an anti-anxiety drug, on the expression of flight behaviors. A CS (95 dB) and a US (0.9 mA) were used. In this experiment, two groups were tested for fear conditioning. On day 1, both groups (n = 8 each) were exposed to 5 CS-alone trials in context A. On days 2 and 3, both groups were conditioned with 5 CS-US trials in context B. On day 4, both groups were exposed to 5 CS-alone trials in context B. Group 1 received saline injections (s.c., 10 ml/kg) before the trials started for all days. Group 2 was injected with saline on day 1 and Diazepam (s.c., 1.2 mg/kg; Fujifilm-Wako, Tokyo, Japan) on days 2–4 before the trials started. All injections were done 30 min before each trial started. The Diazepam solution was prepared by referring to previous reports^43,44^. Diazepam was dissolved in 45% hydroxypropyl-β-cyclodextrin (Fujifilm-Wako) at a concentration of 1.2 mg/ml. Then, before use, it was diluted to a final concentration of 0.12 mg/ml with distilled water. To confirm the antianxiety effect of Diazepam, the elevated plus maze test (10 min) was conducted (saline, n = 4; Diazepam, n = 4), 30 min after saline or Diazepam injection.

The summary of training schedules for these experiments is shown in Fig. 1B.

### Quantification of behaviors

Total motion, number of jumps, and percentage of freezing during each CS presentation were measured. The averaged and total motions of mice were calculated from the difference in the center of mass of the subject silhouette across frames using a custom-made program in MATLAB (The MathWorks. Inc). The 30 A.U. of ‘Motion’ (not ‘total motion’) approximately corresponds to a speed of 25 cm/sec. Jumps were counted manually from video files as an escape behavior. The percentages of freezing were measured by using the transducer. All recorded transducer signals were preprocessed using a 20–500 Hz band pass filter. Duration of immobility longer than one second was defined as freezing. The root mean square amplitude of the transducer signal in time was used to determine the freezing.

### Statistical analysis

For comparisons between groups, day-by-day averages or summations and trial-by-trial numbers were used. In Experiment 3, for comparisons of motion and freezing across days, the number of ‘day 4/day 3’ was defined as ‘across-day ratio’. For the comparison of jumps, ‘day 4/(day 3 + day 4)’ was defined as ‘across-day ratio’. In case the individual did not jump on either day, 0.5 was assigned to the number. Data were analyzed with three-way repeated measures, two-way repeated measures, and one-way ANOVAs, Fisher's exact test, and permutation test. For post-hoc multiple comparisons, t-test with Holm’s correction was used. *P* values were calculated by using MATLAB. *P* values for two-way repeated and one-way ANOVAs were calculated by using the permutation method. Statistical significance was set at *p* < 0.05. Error bars indicate standard error of means (S.E.M.). All statistical results are summarized in the supplementary information.

## Supplementary Information

Supplementary Video 1.

Supplementary Video 2.

Supplementary Video 3.

Supplementary Information 1.

## Data Availability

The datasets generated during and/or analyzed during the current study are available from the corresponding author on reasonable request.
