# Supplementary material for: Multiple factors contribute to flight behaviors during fear conditioning
Source: Sci Rep. 2023 Jun 27;13:10402. doi: 10.1038/s41598-023-37612-0 (PMC10300071; doi:10.1038/s41598-023-37612-0)
Supplement: Supplementary file 4 — Supplementary Information 1. [file 41598_2023_37612_MOESM4_ESM.pdf]

# Multiple factors contribute to flight behaviors during fear conditioning

Takafumi Furuyama[1*], Ayana Imayoshi[1], Toyo Iyobe[1], Munenori Ono[1], Tatsuya Ishikawa[2], Noriyuki Ozaki[2], Nobuo Kato[1], Ryo Yamamoto[1*]

1. Department of Physiology, Kanazawa Medical University, Uchinada, Ishikawa, Japan
2. Department of Functional Anatomy, Graduate School of Medical Science, Kanazawa University, Kanazawa, Ishikawa, Japan

* Corresponding authors:
Ryo Yamamoto
E-mail: ryamamot@kanazawa-med.ac.jp

Takafumi Furuyama
E-mail: tfuruyam@kanazawa-med.ac.jp

# Supplementary information

## Summary of statistical analysis

***Experiment 1***; G1 (n = 10), G2 (n =10), G3 (n = 10)

**Means of motions by trial on day 4 (G1 vs G2 vs G3)**

>Means ± S.E.M. of each condition.

| | t1 | t2 | t3 | t4 | t5 |
|---|---|---|---|---|---|
| G1 | 407.4 ±72.9 | 478.0 ±170.0 | 115.2 ±29.9 | 171.5 ±29.1 | 206.4 ±61.0 |
| G2 | 175.0 ±43.2 | 204.9 ±39.3 | 243.5 ±44.2 | 183.0 ±34.0 | 205.7 ±34.4 |
| G3 | 155.5 ±41.7 | 153.2 ±25.1 | 145.8 ±41.1 | 121.5 ±27.1 | 109.1 ±30.2 |

>2-way repeated ANOVA

| | SS | df | MS | F | p |
|---|---|---|---|---|---|
| group | 481275.3 | 2 | 240637.6 | 3.30 | 0.0448 |
| error | 1967874.5 | 27 | 72884.2 | | |
| days | 347813.4 | 4 | 86953.3 | 3.35 | 0.0085 |
| group * days | 694645.5 | 8 | 86830.7 | 3.34 | 0.0015 |
| error | 2804959.7 | 108 | 25971.8 | | |
| Total | 6296568.3 | 149 | 42258.8 | | |

>Main effects of tone and days (group * days interaction)

| | SS | df | MS | F | p |
|---|---|---|---|---|---|
| group (t1) | 392749.9 | 2 | 196375.0 | 6.61 | 0.0017 |
| error | 802493.1 | 27 | 29722.0 | | |
| group (t2) | 609262.0 | 2 | 304631.0 | 2.94 | 0.0459 |
| error | 2793331.3 | 27 | 103456.7 | | |
| group (t3) | 89861.6 | 2 | 44930.8 | 2.98 | 0.0683 |
| error | 407634.4 | 27 | 15097.6 | | |
| group (t4) | 21397.7 | 2 | 10698.9 | 1.17 | 0.3222 |
| error | 246213.6 | 27 | 9119.0 | | |
| group(t5) | 62649.4 | 2 | 31324.7 | 1.62 | 0.2152 |
| error | 523161.8 | 27 | 19376.4 | | |
| | | | | | |
| trials (G1) | 997125.6 | 4 | 249281.4 | 9.60 | 0.0041 |
| trials (G2) | 28341.7 | 4 | 7085.4 | 0.27 | 0.5793 |
| trials (G3) | 16991.5744 | 4 | 4247.9861 | 0.16 | 0.6772 |
| error | 2804959.7 | 108 | 25971.8 | | |

>Post-hoc t-test with Holm's correction between Groups on trial 1

| pair | nominal level | t | p |
|---|---|---|---|
| G1-G3 | 0.0166 | 3.267 | 0.0030 |

| | | | |
|---|---|---|---|
| G1-G2 | 0.0333 | 3.014 | 0.0056 |
| G2-G3 | 0.05 | 0.253 | 0.8020 |

$S^2$=29722.0,　df=27

>Post-hoc t-test with Holm's correction between Groups on trial 2

| pair | nominal level | t | p |
|---|---|---|---|
| G1-G3 | 0.0166 | 2.258 | 0.0322 |
| G1-G2 | 0.0333 | 1.899 | 0.0683 |
| G2-G3 | 0.05 | 0.359 | 0.3333 |

$S^2$=103456.7,　df=27

**Means of percentages of freezing on day 4　(G1 vs G2 vs G3)**

>Means ± S.E.M.

| | |
|---|---|
| G1 | 45.8 ±4.5 |
| G2 | 30.6 ±6.4 |
| G3 | 71.0 ±3.8 |

>One-way ANOVA

| | SS | df | MS | F | p |
|---|---|---|---|---|---|
| group | 8327.5 | 2 | 4163.7 | 16.51 | 0.0000 |
| error | 6810.0 | 27 | 252.2 | | |
| total | 15137.5 | 29 | | | |

>Post-hoc t-test with Holm's correction

| pair | nominal level | t | p |
|---|---|---|---|
| G3-G2 | 0.0166 | 5.688 | 0.0000 |
| G3-G1 | 0.0333 | 3.548 | 0.0014 |
| G1-G2 | 0.05 | 2.140 | 0.0425 |

$S^2$=252.2,　df=27

**Means of total numbers of jumps on day 4 (G1 vs G2 vs G3)**

>Means ± S.E.M.

| | |
|---|---|
| G1 | 3.4 ±1.5 |
| G2 | 0.9 ±0.5 |
| G3 | 0.0 ±0.0 |

>One-way ANOVA

| | SS | df | MS | F | p |
|---|---|---|---|---|---|
| group | 62.1 | 2 | 31.0 | 3.72 | 0.0258 |
| error | 225.3 | 27 | 8.3 | | |
| total | 287.4 | 29 | | | |

>Post-hoc t-test with Holm’s correction

| pair | nominal level | t | p |
|---|---|---|---|
| G1-G3 | 0.0166 | 2.632 | 0.0139 |
| G1-G2 | 0.0333 | 1.935 | 0.0635 |
| G2-G3 | 0.05 | 0.697 | 0.4920 |

$S^2$=8.3,  df=27

**Ratios of (jumps during CS / total jumps) on day 4 (G1 vs G2)**

> Fisher's exact test

G1, 6/40; G2, 9/18; p = 0.0087

*Experiment 2*; G1 (n = 11), G2 (n =10), G3 (n = 10), G4 (n = 10)

**Means of total motions by day (G1 vs G2 vs G3 vs G4)**

>Means ± S.E.M. of each condition.

| | Day1 | Day2 | Day3 | Day4 |
|---|---|---|---|---|
| G1 | 465.0 ±33.2 | 235.4 ±26.2 | 449.3 ±109.9 | 291.6 ±72.7 |
| G2 | 424.4 ±75.0 | 246.6 ±37.9 | 390.2 ±62.5 | 161.9 ±22.0 |
| G3 | 546.8 ±25.4 | 145.2 ±16.9 | 191.8 ±51.5 | 153.5 ±39.2 |
| G4 | 472.1 ±31.9 | 142.4 ±14.7 | 100.3 ±14.3 | 100.8 ±20.5 |

>3-way repeated ANOVA (factors CS, US, and days)

| | SS | df | MS | F | p |
|---|---|---|---|---|---|
| CS | 421122.3 | 1 | 421122.3 | 9.10 | 0.0046 |
| US | 123763.0 | 1 | 123763.0 | 2.68 | 0.1101 |
| CS * US | 8.2 | 1 | 8.2 | 0.00 | 0.9894 |
| error | 1711356.0 | 37 | 46252.9 | | |
| days | 2337964.7 | 3 | 779321.6 | 42.13 | 0.0000 |
| US * days | 53543.3 | 3 | 17847.8 | 0.96 | 0.4144 |
| CS * days | 586194.0 | 3 | 195398.0 | 10.56 | 0.0000 |
| CS * US * days | 21332.5 | 3 | 7110.8 | 0.38 | 0.7676 |
| error | 2053446.4 | 111 | 18499.5 | | |
| Total | 7308730.3 | 163 | | | |

>Main effects of CS and days (CS * days interaction)

| | SS | df | MS | F | p |
|---|---|---|---|---|---|
| CS (day1) | 42845.8 | 1 | 42845.8 | 2.04 | 0.1616 |
| error | 777117.7 | 37 | 21003.2 | | |
| CS (day2) | 96629.2 | 1 | 96629.2 | 14.30 | 0.0006 |
| error | 250090.5 | 37 | 6759.2 | | |
| CS (day3) | 766322.4 | 1 | 766322.4 | 14.64 | 0.0005 |
| error | 1936169.2 | 37 | 52328.9 | | |
| CS (day4) | 101518.9 | 1 | 101518.9 | 4.69 | 0.0369 |
| error | 801425.0 | 37 | 21660.1 | | |
| | | | | | |
| days (CS 95dB) | 813715.9 | 3 | 271238.6 | 14.66 | 0.0000 |
| days (CS 75dB) | 2110442.9 | 3 | 703481.0 | 38.03 | 0.0000 |
| error | 801425.0 | 111 | 18499.5 | | |

**Means of percentages of freezing by day (G1 vs G2 vs G3 vs G4)**

>Means ± S.E.M. of each condition.

| | Day1 | Day2 | Day3 | Day4 |
|---|---|---|---|---|

| | | | | |
|---|---|---|---|---|
| G1 | 7.5 ±1.9 | 37.8 ±5.1 | 50.1 ±5.5 | 57.4 ±6.1 |
| G2 | 12.6 ±4.3 | 37.4 ±3.9 | 47.3 ±4.3 | 67.3 ±4.3 |
| G3 | 1.8 ±0.6 | 48.3 ±4.2 | 68.6 ±5.5 | 70.4 ±5.0 |
| G4 | 4.1 ±1.2 | 43.6 ±2.3 | 71.9 ±4.6 | 73.0 ±5.2 |

>3-way repeated ANOVA (factors CS, US, and days)

| | SS | df | MS | F | p |
|---|---|---|---|---|---|
| CS | 2642.7 | 1 | 2642.7 | 7.49 | 0.0095 |
| US | 149.4 | 1 | 149.4 | 0.42 | 0.5209 |
| CS * US | 43.9 | 1 | 43.9 | 0.12 | 0.7310 |
| error | 13060.3 | 37 | 353.0 | 3.34 | |
| days | 89190.8 | 3 | 29730.3 | 213.06 | 0.0000 |
| US * days | 458.7 | 3 | 152.9 | 1.10 | 0.3523 |
| CS * days | 4240.4 | 3 | 1413.5 | 10.13 | 0.0000 |
| CS * US * days | 255.0 | 3 | 85.0 | 0.61 | 0.6099 |
| error | 15489.2 | 111 | 139.5 | | |
| Total | 125530.4 | 163 | | | |

>Main effects of CS and days (CS * days interaction)

| | SS | df | MS | F | p |
|---|---|---|---|---|---|
| CS (day1) | 519.1 | 1 | 519.1 | 8.87 | 0.0051 |
| error | 2165.6 | 37 | 58.5 | | |
| CS (day2) | 711.9 | 1 | 711.9 | 4.17 | 0.0483 |
| error | 6318.5 | 37 | 170.8 | | |
| CS (day3) | 4754.0 | 1 | 4754.0 | 18.25 | 0.0001 |
| error | 9640.3 | 37 | 260.5 | | |
| CS (day4) | 898.0 | 1 | 898.0 | 3.19 | 0.0823 |
| error | 10425.0 | 37 | 281.8 | | |
| | | | | | |
| days (CS 95dB) | 30191.4 | 3 | 10063.8 | 20.582 | 0.0000 |
| days (CS 75dB) | 63239.8 | 3 | 21079.9 | 33.111 | 0.0000 |
| error | 10425.0 | 111 | 139.5422 | | |

**Means of total numbers of jumps by day (G1 vs G2 vs G3 vs G4)**

>Means ± S.E.M. of each condition.

| | Day1 | Day2 | Day3 | Day4 |
|---|---|---|---|---|
| G1 | 0.2 ±0.1 | 0.1 ±0.1 | 6.5 ±2.6 | 3.6 ±1.8 |
| G2 | 0.3 ±0.2 | 1.0 ±0.6 | 6.6 ±2.0 | 2.7 ±1.0 |
| G3 | 0.2 ±0.2 | 0.1 ±0.1 | 2.6 ±1.3 | 2.4 ±1.1 |

| | | | | |
|---|---|---|---|---|
| G4 | 0.0 ±0.0 | 0.0 ±0.0 | 0.2 ±0.1 | 0.5 ±0.3 |

>3-way repeated ANOVA (factors CS, US, and days)

| | SS | df | MS | F | p |
|---|---|---|---|---|---|
| CS | 143.2 | 1 | 143.2 | 7.51 | 0.0094 |
| US | 12.2 | 1 | 12.2 | 0.64 | 0.4288 |
| CS * US | 15.0 | 1 | 15.0 | 0.78 | 0.3828 |
| error | 705.3 | 37 | 19.1 | | |
| days | 401.1 | 3 | 133.7 | 12.45 | 0.0000 |
| US * days | 23.1 | 3 | 7.7 | 0.72 | 0.5421 |
| CS * days | 158.7 | 3 | 52.9 | 4.92 | 0.0030 |
| CS * US * days | 6.9 | 3 | 2.3 | 0.21 | 0.8893 |
| error | 1192.5 | 111 | 10.7 | | |
| Total | 2658.0 | 163 | | | |

>Main effects of CS and days (CS * days interaction)

| | SS | df | MS | F | p |
|---|---|---|---|---|---|
| CS (day1) | 0.2 | 1 | 0.2 | 0.81 | 0.3739 |
| error | 9.3 | 37 | 0.3 | | |
| CS (day2) | 2.5 | 1 | 2.5 | 2.75 | 0.1057 |
| error | 33.8 | 37 | 0.9 | | |
| CS (day3) | 269.0 | 1 | 269.0 | 7.67 | 0.0087 |
| error | 1297.1 | 37 | 35.1 | | |
| CS (day4) | 30.2 | 1 | 30.2 | 2.00 | 0.1657 |
| error | 557.5 | 37 | 15.1 | | |
| | | | | | |
| days (CS 95dB) | 522.5 | 3 | 174.2 | 16.21 | 0.0000 |
| days (CS 75dB) | 37.3 | 3 | 12.4 | 1.16 | 0.3284 |
| error | 557.5 | 111 | 10.7 | | |

***Experiment 3***; G1 (n = 10), G2 (n =10)

**Means of total motions by day (G1 vs G2)**

>Means ± S.E.M. of each condition.

| | Day1 | Day2 | Day3 | Day4 |
|---|---|---|---|---|
| G1 | 476.9 ±54.5 | 134.1 ±23.5 | 131.3 ±32.4 | 288.6 ±78.0 |
| G2 | 594.0 ±45.3 | 227.2 ±24.3 | 261.5 ±48.9 | 110.5 ±32.4 |

>2-way repeated ANOVA (factor; tone and days)

| | SS | df | MS | F | p |
|---|---|---|---|---|---|
| tone | 32926.7 | 1 | 32926.7 | 0.74 | 0.4066 |
| error | 803517.7 | 18 | 44639.9 | | |
| days | 1771732.2 | 3 | 590577.4 | 45.43 | 0.0000 |
| tone * days | 322409.9 | 3 | 107470.0 | 8.27 | 0.0000 |
| error | 702034.9 | 54 | 13000.6 | | |
| total | 3632621.4 | 79 | | | |

>Main effects of tone and days (tone * days interaction)

| | SS | df | MS | F | p |
|---|---|---|---|---|---|
| tone (day1) | 68562.0 | 1 | 68562.0 | 2.73 | 0.1220 |
| error | 451846.5 | 18 | 25102.6 | | |
| tone (day2) | 43365.9 | 1 | 43365.9 | 7.60 | 0.0139 |
| error | 102644.5 | 18 | 5702.5 | | |
| tone (day3) | 84759.2 | 1 | 84759.2 | 4.93 | 0.0330 |
| error | 309757.5 | 18 | 17208.7 | | |
| tone (day4) | 158649.5 | 1 | 158649.5 | 4.45 | 0.0467 |
| error | 641304.1 | 18 | 35628.0 | | |
| | | | | | |
| days (G1) | 802744.1 | 3 | 267581.5 | 20.582 | 0.0000 |
| days (G2) | 1291398.0 | 3 | 430466.0 | 33.111 | 0.0000 |
| error | 702034.9 | 54 | 13000.6 | | |

**Means of percentages of freezing by day (G1 vs G2)**

>Means ± S.E.M. of each condition.

| | Day1 | Day2 | Day3 | Day4 |
|---|---|---|---|---|
| G1 | 6.0 ±2.1 | 52.6 ±5.4 | 70.8 ±6.3 | 50.3 ±6.9 |
| G2 | 4.3 ±2.9 | 34.0 ±5.5 | 51.6 ±7.1 | 76.1 ±5.7 |

>2-way repeated ANOVA (factor; tone and days)

| | SS | df | MS | F | p |
|---|---|---|---|---|---|
| tone | 234.6 | 1 | 234.6 | 0.31 | 0.5896 |

| | | | | | |
|---|---|---|---|---|---|
| error | 13745.5 | 18 | 763.6 | | |
| days | 43436.2 | 3 | 14478.7 | 96.76 | 0.0000 |
| tone * days | 6681.0 | 3 | 2227.0 | 14.88 | 0.0000 |
| error | 8080.0 | 54 | 149.6 | | |
| total | 72177.4 | 79 | | | |

>Main effects of tone and days (tone * days interaction)

| | SS | df | MS | F | p |
|---|---|---|---|---|---|
| tone (day1) | 14.5 | 1 | 114.5 | 0.23 | 0.6918 |
| error | 1154.1 | 18 | 64.1 | | |
| tone (day2) | 1729.8 | 1 | 1729.8 | 5.80 | 0.0279 |
| error | 5368.4 | 18 | 298.2 | | |
| tone (day3) | 1843.2 | 1 | 1843.2 | 4.11 | 0.0628 |
| error | 8064.0 | 18 | 448.0 | | |
| tone (day4) | 3328.2 | 1 | 3328.2 | 8.28 | 0.0111 |
| error | 7239.0 | 18 | 402.2 | | |
| | | | | | |
| days (G1) | 222724.7 | 3 | 7574.9 | 50.62 | 0.0000 |
| days (G2) | 27392.6 | 3 | 9130.9 | 61.02 | 0.0000 |
| error | 8080.0 | 54 | 149.6 | | |

**Means of total numbers of jumps by day (G1 vs G2)**

>Means ± S.E.M. of each condition.

| | Day1 | Day2 | Day3 | Day4 |
|---|---|---|---|---|
| G1 | 0.1 ±0.1 | 0.2 ±0.1 | 0.9 ±0.5 | 3.8 ±1.2 |
| G2 | 0.2 ±0.2 | 0.3 ±0.2 | 2.7 ±1.6 | 1.7 ±1.1 |

>2-way repeated ANOVA (factor; tone and days)

| | SS | df | MS | F | p |
|---|---|---|---|---|---|
| tone | 0.0 | 1 | 0.0 | 0.00 | 0.9942 |
| error | 200.2 | 18 | 11.1 | | |
| days | 95.2 | 3 | 31.7 | 5.38 | 0.0015 |
| tone * days | 38.3 | 3 | 12.8 | 2.17 | 0.0921 |
| error | 318.7 | 54 | 5.9 | | |
| total | 652.5 | 79 | 8.3 | | |

**Means of 'across-day ratio' (G1 vs G2)**

>Means ± S.E.M. of each condition.

| | motion | freezing | jumps |
|---|---|---|---|
| G1 | 2.67 ±0.59 | 0.71 ±0.08 | 0.66 ±0.10 |

| | | | |
|---|---|---|---|
| G2 | 0.39 ±0.08 | 1.60 ±0.15 | 0.33 ±0.08 |

>Permutation test

| pair | p |
|---|---|
| Motion | 0.0000 |
| Freezing | 0.0000 |
| Jumps | 0.0216 |

***Experiment 4***; G1 (n = 8), G2 (n =8)

**Means of total motions by day (G1 vs G2)**

>Means ± S.E.M. of each condition.

| | Day1 | Day2 | Day3 | Day4 |
|---|---|---|---|---|
| G1 | 540.7 ±39.4 | 204.1 ±25.2 | 331.4 ±91.9 | 148.6 ±29.2 |
| G2 | 606.7 ±37.7 | 225.0 ±24.1 | 132.5 ±25.6 | 111.2 ±22.0 |

>2-way repeated ANOVA (factor; schedule and days)

| | SS | df | MS | F | p |
|---|---|---|---|---|---|
| schedule | 22280.6 | 1 | 22280.6 | 0.77 | 0.3929 |
| error | 407307.9 | 14 | 29093.4 | | |
| days | 1842227.4 | 3 | 614075.8 | 62.67 | 0.0000 |
| schedule * days | 160723.9 | 3 | 53574.6 | 5.47 | 0.0029 |
| error | 411539.5 | 42 | 9798.6 | | |
| total | 2844079.3 | 63 | 45144.1 | | |

>Main effects of tone and days (schedule * days interaction)

| | SS | df | MS | F | p |
|---|---|---|---|---|---|
| schedule (day1) | 17426.9 | 1 | 17426.9 | 1.47 | 0.2462 |
| error | 166350.0 | 14 | 11882.1 | | |
| schedule (day2) | 1761.6 | 1 | 1761.6 | 0.36 | 0.5451 |
| error | 68251.1 | 14 | 4875.1 | | |
| schedule (day3) | 158230.7 | 1 | 158230.7 | 4.35 | 0.0483 |
| error | 509375.0 | 14 | 36383.9 | | |
| schedule (day4) | 5585.2 | 1 | 5585.2 | 1.04 | 0.3251 |
| error | 74871.3 | 14 | 5347.9 | | |
| | | | | | |
| days (G1) | 727065.7 | 3 | 242355.2 | 24.73 | 0.0000 |
| days (G2) | 1275885.6 | 3 | 425295.2 | 43.40 | 0.0000 |
| error | 411539.5 | 42 | 9798.6 | | |

**Means of percentages of freezing by day (G1 vs G2)**

>Means ± S.E.M. of each condition.

| | Day1 | Day2 | Day3 | Day4 |
|---|---|---|---|---|
| G1 | 5.4 ±1.9 | 38.4 ±4.4 | 49.6 ±5.8 | 60.3 ±5.2 |
| G2 | 2.6 ±0.6 | 33.0 ±3.9 | 64.1 ±5.3 | 69.0 ±5.2 |

>2-way repeated ANOVA (factor; schedule and days)

| | SS | df | MS | F | p |
|---|---|---|---|---|---|
| schedule | 228.8 | 1 | 228.8 | 0.81 | 0.3853 |

| | | | | | |
|---|---|---|---|---|---|
| error | 3975.3 | 14 | 284.0 | | |
| days | 35286.4 | 3 | 11762.1 | 105.19 | 0.0000 |
| schedule * days | 1064.3 | 3 | 354.8 | 3.17 | 0.0368 |
| error | 4696.5 | 42 | 111.8 | | |
| total | 45251.4 | 63 | 718.3 | | |

>Main effects of tone and days (tone * days interaction)

| | SS | df | MS | F | p |
|---|---|---|---|---|---|
| schedule (day1) | 30.3 | 1 | 30.3 | 1.91 | 0.2530 |
| error | 221.8 | 14 | 15.8 | | |
| schedule (day2) | 115.6 | 1 | 115.6 | 0.84 | 0.3827 |
| error | 1935.9 | 14 | 138.3 | | |
| schedule (day3) | 841.0 | 1 | 841.0 | 3.38 | 0.0917 |
| error | 3480.8 | 14 | 248.6 | | |
| schedule (day4) | 306.3 | 1 | 306.3 | 1.41 | 0.2573 |
| error | 3033.5 | 14 | 216.7 | | |
| | | | | | |
| days (G1) | 13552.6 | 3 | 4517.5 | 40.40 | 0.0000 |
| days (G2) | 22798.1 | 3 | 7599.4 | 67.96 | 0.0000 |
| error | 4696.5 | 42 | 111.8 | | |

**Means of total numbers of jumps by day (G1 vs G2)**

>Means ± S.E.M. of each condition.

| | Day1 | Day2 | Day3 | Day4 |
|---|---|---|---|---|
| G1 | 0.0 ±0.0 | 0.0 ±0.0 | 4.4 ±2.2 | 1.8 ±0.9 |
| G2 | 0.0 ±0.0 | 0.1 ±0.1 | 0.0 ±0.0 | 0.0 ±0.0 |

>2-way repeated ANOVA (factor; tone and days)

| | SS | df | MS | F | p |
|---|---|---|---|---|---|
| schedule | 36.0 | 1 | 36.0 | 5.39 | 0.0280 |
| error | 93.4 | 14 | 6.7 | | |
| days | 49.8 | 3 | 16.6 | 3.19 | 0.0235 |
| schedule * days | 52.9 | 3 | 17.6 | 3.38 | 0.0119 |
| error | 218.8 | 42 | 5.2 | | |
| total | 450.9 | 63 | 7.2 | | |

>Main effects of tone and days (tone * days interaction)

| | SS | df | MS | F | p |
|---|---|---|---|---|---|
| schedule (day1) | 0.0 | 1 | 0.0 | 0.00 | 0.0000 |
| error | 0.0 | 14 | 0.0 | | |

| | | | | | |
|---|---|---|---|---|---|
| schedule (day2) | 0.1 | 1 | 0.1 | 1.00 | 1.0000 |
| error | 0.9 | 14 | 0.1 | | |
| schedule (day3) | 76.6 | 1 | 76.6 | 4.03 | 0.1964 |
| error | 265.9 | 14 | 18.99 | | |
| schedule (day4) | 12.3 | 1 | 12.3 | 3.77 | 0.1907 |
| error | 45.5 | 14 | 3.3 | | |
| | | | | | |
| days (G1) | 102.6 | 3 | 34.2 | 6.56 | 0.0267 |
| days (G2) | 0.1 | 3 | 0.0 | 0.01 | 0.0927 |
| error | 218.8 | 42 | 5.2 | | |

***Experiment 5***; G1 (n = 8), G2 (n =8)

**Means of total motions by day (G1 vs G2)**

>Means ± S.E.M. of each condition.

| | Day1 | Day2 | Day3 | Day4 |
|---|---|---|---|---|
| G1 | 697.0 ±25.3 | 279.5 ±36.3 | 518.2 ±101.6 | 268.9 ±42.7 |
| G2 | 656.9 ±26.7 | 222.7 ±21.7 | 293.8 ±38.9 | 135.6 ±23.4 |

>2-way repeated ANOVA (factor; drug and days)

| | SS | df | MS | F | p |
|---|---|---|---|---|---|
| drug | 206710.2 | 1 | 206710.2 | 9.71 | 0.0073 |
| error | 298124.8 | 14 | 21294.6 | | |
| days | 2191966.7 | 3 | 730655.6 | 45.63 | 0.0000 |
| drug * days | 85256.3 | 3 | 28418.8 | 1.77 | 0.1666 |
| error | 672528.2 | 42 | 16012.6 | | |
| total | 3454586.2 | 63 | 54834.7 | | |

**Means of percentages of freezing by day (G1 vs G2)**

>Means ± S.E.M. of each condition.

| | Day1 | Day2 | Day3 | Day4 |
|---|---|---|---|---|
| G1 | 0.4 ±0.3 | 25.1 ±4.5 | 37.8 ±5.8 | 44.8 ±8.0 |
| G2 | 1.5 ±0.6 | 46.1 ±4.0 | 51.0 ±5.3 | 66.8 ±6.8 |

>2-way repeated ANOVA (factor; tone and days)

| | SS | df | MS | F | p |
|---|---|---|---|---|---|
| drug | 3291.9 | 1 | 3291.9 | 14.28 | 0.0025 |
| error | 3228.5 | 14 | 230.6 | | |
| days | 26821.7 | 3 | 8940.6 | 44.29 | 0.0000 |
| drug * days | 1115.4 | 3 | 371.8 | 1.84 | 0.1506 |
| error | 8477.7 | 42 | 201.8 | | |
| total | 42935.1 | 63 | 681.5 | | |

**Means of total numbers of jumps by day (G1 vs G2)**

>Means ± S.E.M. of each condition.

| | Day1 | Day2 | Day3 | Day4 |
|---|---|---|---|---|
| G1 | 0.0 ±0.0 | 0.6 ±0.4 | 6.3 ±2.1 | 1.6 ±0.9 |
| G2 | 0.0 ±0.0 | 0.0 ±0.0 | 1.3 ±1.1 | 0.8 ±0.5 |

>2-way repeated ANOVA (factor; tone and days)

| | SS | df | MS | F | p |
|---|---|---|---|---|---|

| | | | | | |
|---|---|---|---|---|---|
| drug | 42.3 | 1 | 42.3 | 4.59 | 0.0444 |
| error | 129.0 | 14 | 9.2 | | |
| days | 138.9 | 3 | 46.3 | 7.99 | 0.0000 |
| drug * days | 62.4 | 3 | 20.8 | 3.59 | 0.0139 |
| error | 243.3 | 42 | 5.8 | | |
| total | 615.8 | 63 | 9.8 | | |

>Main effects of tone and days (tone * days interaction)

| | SS | df | MS | F | p |
|---|---|---|---|---|---|
| drug (day1) | 0 | 1 | 0 | 0 | 0 |
| error | 0 | 14 | 0 | | |
| drug (day2) | 1.6 | 1 | 1.6 | 2.22 | 0.4673 |
| error | 9.9 | 14 | 0.7 | | |
| drug (day3) | 100.0 | 1 | 100.0 | 4.59 | 0.0338 |
| error | 305.0 | 14 | 21.8 | | |
| drug (day4) | 3.1 | 1 | 3.1 | 0.75 | 0.5146 |
| error | 45.5 | 14 | 3.3 | | |
| | | | | | |
| days (G1) | 192.3 | 3 | 64.1 | 11.06 | 0.0014 |
| days (G2) | 9.0 | 3 | 3.0 | 0.52 | 0.1436 |
| error | 243.3 | 42 | 5.8 | | |

**Elevated plus maze test**

>Permutation test

| pair | p |
|---|---|
| % time spent in open arm | 0.0242 |
| Distance traveled | 0.3442 |

# Supplementary figures

S1

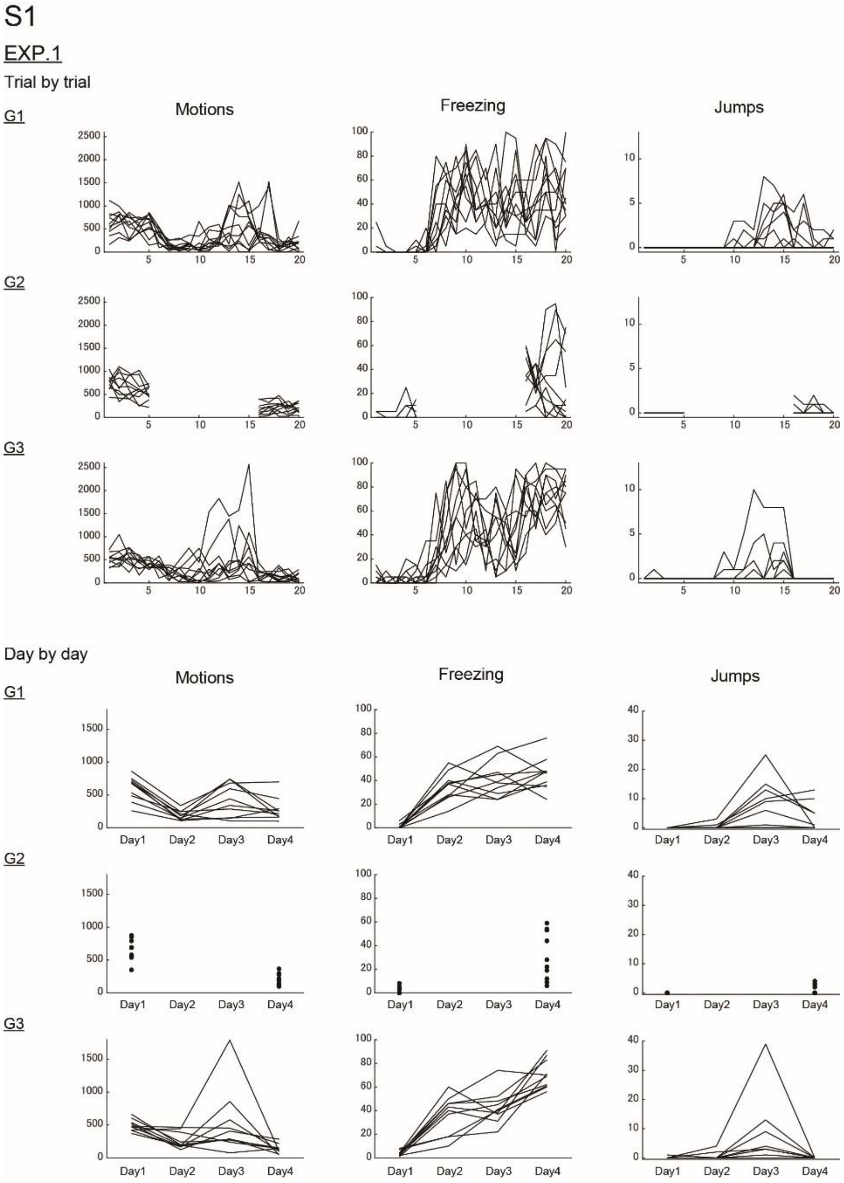


Fig. S1 All individual plots for Experiment 1 are shown. Trial-by-trial plots for motions, freezing, and jumps of three groups are presented in the upper half of the figure. Day-by-day plots for motions, freezing, and jumps of three groups are presented in the lower half of the figure.

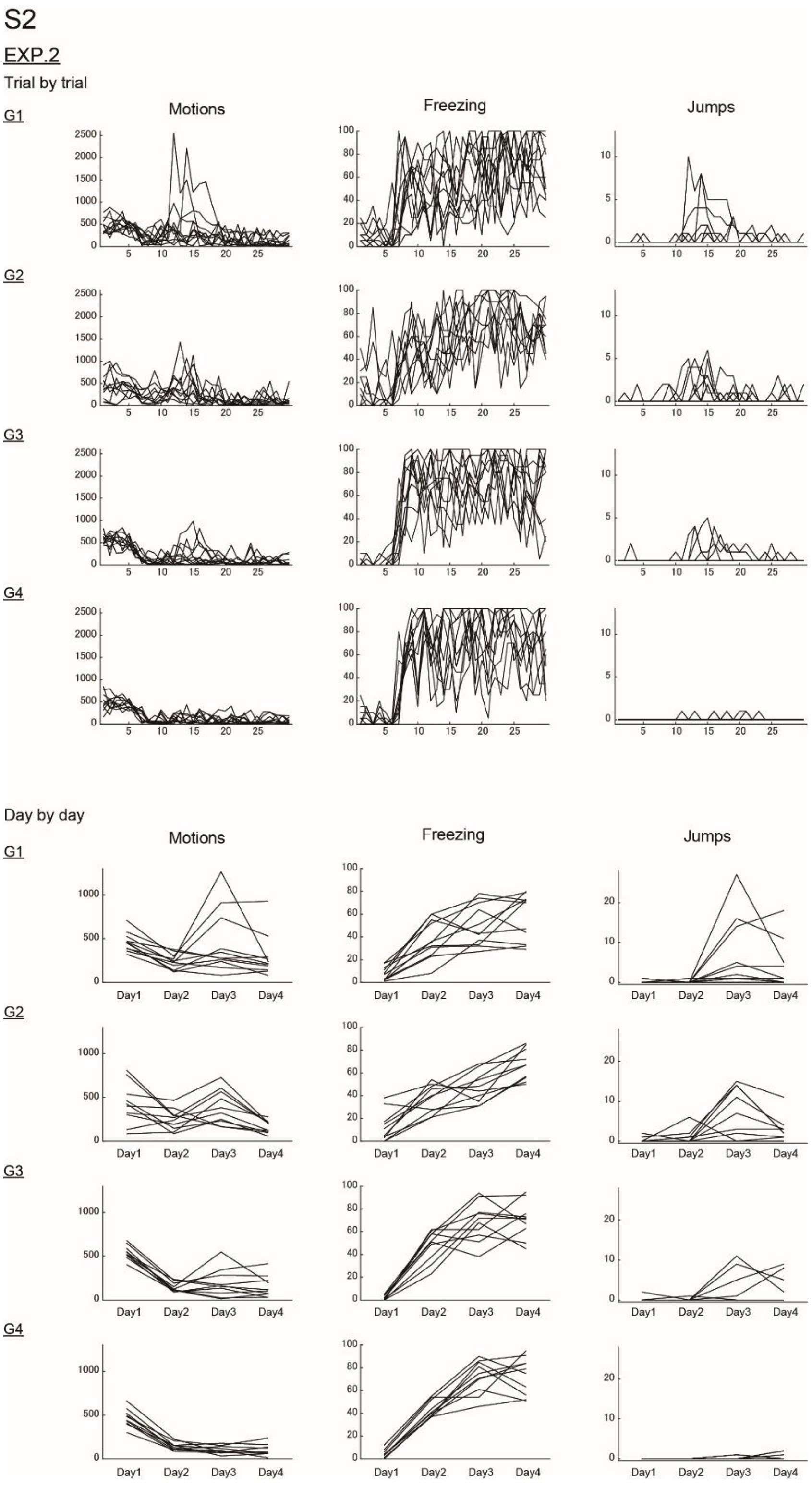


Fig. S2 All individual plots for Experiment 2 are shown. Trial-by-trial plots for motions, freezing, and jumps of four groups are presented in the upper half of the figure. Day-by-day plots for motions, freezing, and jumps of four groups are presented in the lower half of the figure.

S3

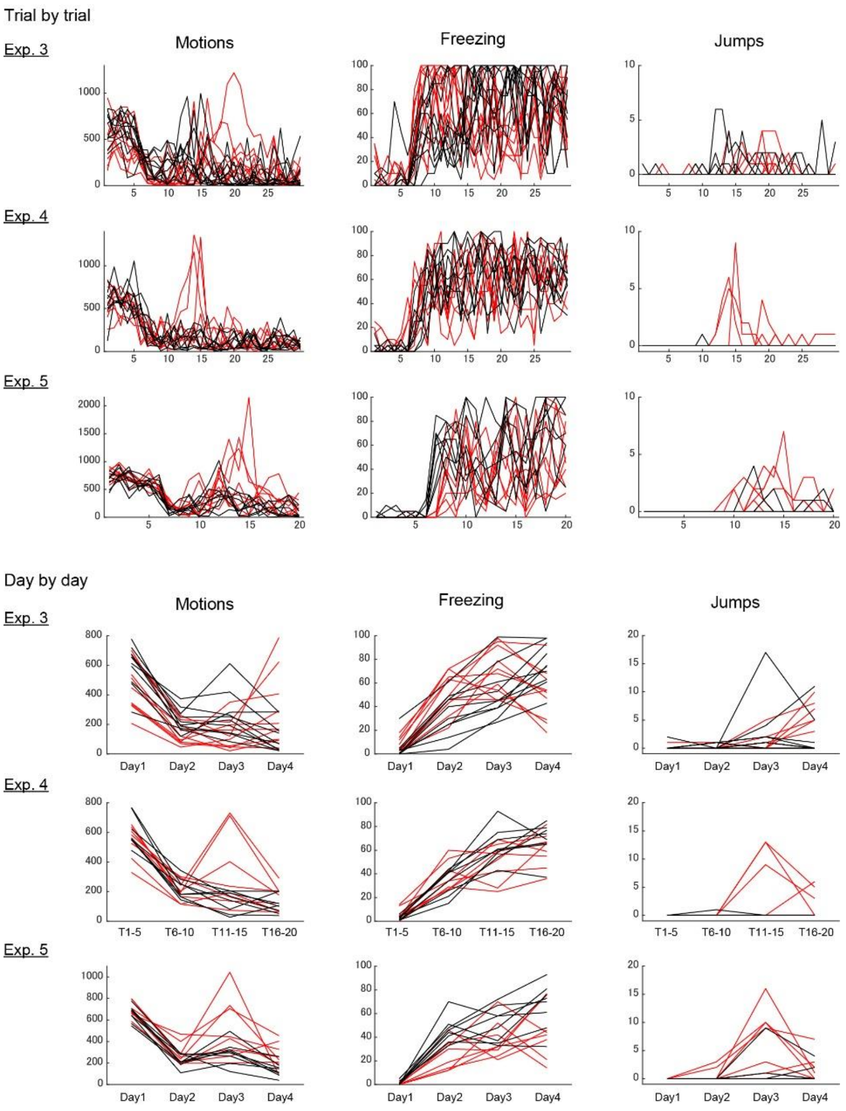


Fig. S3 All individual plots for Experiments 3-5 are shown. Black plots indicate results from Group 1 and red plots indicate results from Group 2. Trial-by-trial plots for motions, freezing, and jumps of four groups are presented in the upper half of the figure. Day-by-day plots for motions, freezing, and jumps of four groups are presented in the lower half of the figure.

S4

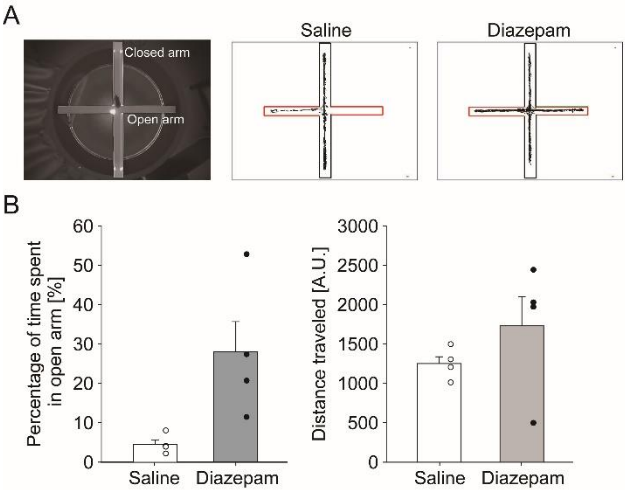


Fig. S4 Effect of Diazepam (1.2 mg/kg) was tested in the elevated plus maze test. (A) *Left*; a picture of the elevated plus maze. *Middle*; a trajectory of a saline control subject is shown. Red arms represent open arms. *Right*; a trajectory of a Diazepam-treated subject is shown. (B) Summary diagrams of the percentage of time spent in open arms and the distance traveled. With Diazepam, subjects spent a much longer time in open arms than the control subjects, while the total distance traveled was unchanged.

**Supplementary movie legend**

M1. Representative jump behaviors are shown. A subject started jumping up on the tone presentation.

M2. A representative darting is shown. A subject darted by the tone presentation.

M3. A representative freezing is shown. A subject exhibited freezing during the tone presentation.
